# Deletion of an X-Inactivation Boundary Disrupts Adjacent Gene Silencing

**DOI:** 10.1371/journal.pgen.1003952

**Published:** 2013-11-21

**Authors:** Lindsay M. Horvath, Nan Li, Laura Carrel

**Affiliations:** Department of Biochemistry and Molecular Biology, Pennsylvania State College of Medicine, Hershey, Pennsylvania, United States of America; University of Pennsylvania, United States of America

## Abstract

In mammalian females, genes on one X are largely silenced by X-chromosome inactivation (XCI), although some “escape” XCI and are expressed from both Xs. Escapees can closely juxtapose X-inactivated genes and provide a tractable model for assessing boundary function at epigenetically regulated loci. To delimit sequences at an XCI boundary, we examined female mouse embryonic stem cells carrying X-linked BAC transgenes derived from an endogenous escape locus. Previously we determined that large BACs carrying escapee *Kdm5c* and flanking X-inactivated transcripts are properly regulated. Here we identify two lines with truncated BACs that partially and completely delete the distal *Kdm5c* XCI boundary. This boundary is not required for escape, since despite integrating into regions that are normally X inactivated, transgenic *Kdm5c* escapes XCI, as determined by RNA FISH and by structurally adopting an active conformation that facilitates long-range preferential association with other escapees. Yet, XCI regulation is disrupted in the transgene fully lacking the distal boundary; integration site genes up to 350 kb downstream of the transgene now inappropriately escape XCI. Altogether, these results reveal two genetically separable XCI regulatory activities at *Kdm5c*. XCI escape is driven by a dominant element(s) retained in the shortest transgene that therefore lies within or upstream of the *Kdm5c* locus. Additionally, the distal XCI boundary normally plays an essential role in preventing nearby genes from escaping XCI.

## Author Summary

Early in mammalian female development, one X chromosome is largely silenced to equalize X-linked gene expression between the sexes. Nevertheless, some genes “escape” this silencing and therefore are expressed from both X chromosomes. Understanding how these escape genes are regulated, particularly when they closely juxtapose silenced genes, may give important insight into regulatory transitions throughout the genome. To evaluate sequences that are essential for appropriate inactive X expression we analyzed large transgenes that integrated on the X chromosome in mouse embryonic stem cells. Transgenes that include an escape gene, *Kdm5c*, but lack all or part of the downstream sequences, including the X-inactivation boundary, still escape X inactivation. Nevertheless, downstream genes at the transgene insertion site are misregulated and now inappropriately escape X inactivation as well. These data identify two important regulatory activities at this locus. First, sequences retained within the truncated transgene are sufficient to direct the *Kdm5c* gene to escape X inactivation. Further, we have uncovered a function for an X-inactivation boundary in protecting adjacent genes from escape.

## Introduction

Recent annotation of the human and mouse genomes has revealed chromosome domains that are distinguished by sequence and gene content, regulatory-factor binding, replication dynamics, chromatin composition, or nuclear location. Many of these domains overlap and can functionally segregate active and inactive transcripts [1]–[4]. What regulates such extensive genome compartmentalization is not fully understood. Intriguingly, many boundaries share common features including opposing chromatin marks, active transcription, or binding by the CCCTC binding factor, CTCF [1], [4]–[6]. Whether these elements are essential for segregating domains has not been thoroughly examined, yet boundary deletion can lead to misregulation (e.g. [7]).

An interesting example of partitioned, closely juxtaposed, active and inactive transcripts is found on one X chromosome in female mammals. This X is largely silenced during early embryonic development in order to balance dosage between the sexes. X-chromosome inactivation (XCI) is mediated by the *cis*-limited action of *Xist*, a structural RNA that coats the X chromosome and recruits inactive chromatin modifiers [8]. Nevertheless, XCI is not chromosome-wide, as some genes “escape” inactivation [9]. Current understanding of how genes escape XCI on an otherwise silenced chromosome is incomplete, but the answer may reveal novel insights about regulatory sequences not only at XCI boundaries but also at other expression transitions throughout the genome.

Escape and X-inactivated genes are epigenetically and structurally distinct [9]. Escape genes are depleted in *Xist* RNA and promoters are marked by active histone modifications and lack silent epigenetic marks associated with X-inactivated transcripts (e.g. [10]–[12]). However, long-range regulation is likely involved, as many escape genes, particularly in humans, are physically clustered [13], [14]. Further supporting this idea, unique sequence composition distinguishes these domains relative to the rest of the X [15], [16]. Distant escapees also frequently interact on the inactive X [17] and can be spatially separated from silent inactive X regions [18].

To functionally delimit sequences sufficient to confer XCI escape, we previously developed a transgene approach in female mouse embryonic stem (ES) cells, a well established *ex vivo* XCI model [19]. X-linked BAC transgene lines were isolated that carry the escapee *Kdm5c* (previously *Jarid1c*) that encodes a histone H3K4 demethylase [20], [21]. The BACs also included an adjacent long non-coding RNA (lncRNA) *AK148627* that escapes XCI [14], [22] and flanking X-inactivated genes [16], [19], [23]. Endogenous expression patterns examined were maintained including transgenic *Kdm5c* (*Kdm5c-tg*) escape at four ectopic X-chromosome locations. Therefore, these BACs must include sequences necessary for *Kdm5c* to escape XCI. What features at this locus direct XCI escape? Plausible candidates include CTCF and the *AK148627* lncRNA, as both CTCF and lncRNAs are found at a number of XCI boundaries [14], [22], [24]. Further, such elements are enriched at other boundaries throughout the genome (e.g. [1], [4], [5]), and can function to regulate adjacent genes in *cis* [25], [26]. Intriguing associations notwithstanding, both candidates lack functional validation. To better understand the role of boundary sequences in inactive X regulation we now extend our analysis of *Kdm5c* BAC transgenes. We further narrow sequences necessary for XCI escape and identify a novel role for XCI boundary sequences in regulating inactive X expression.

## Results

### An X-linked transgene deletion series

**Figure 1:**
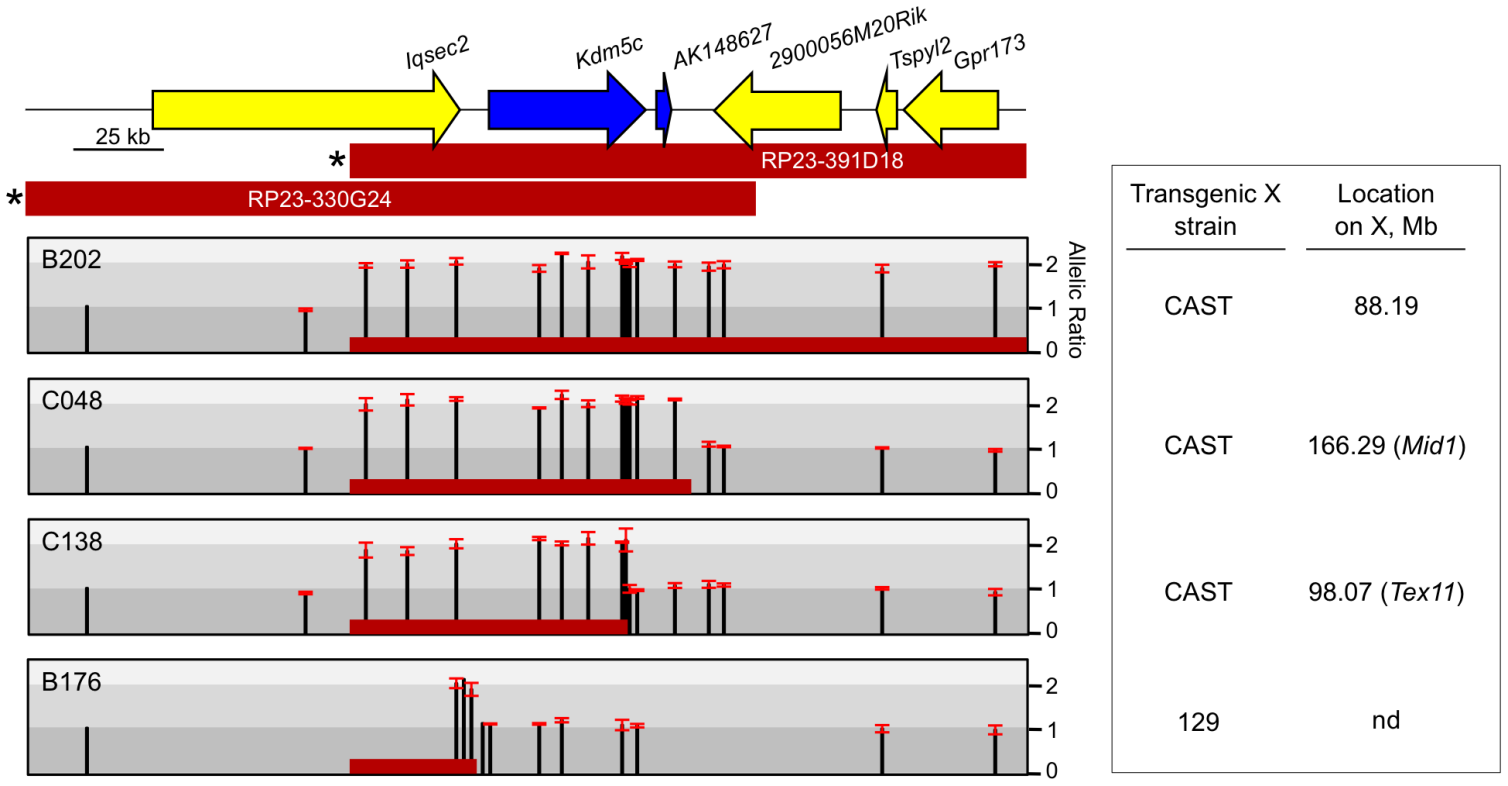
Mapping transgene deletion breakpoints. Endogenous *Kdm5c* locus at chrX:148,557,326-148,807,402 (mm9). Escapees are blue. BACs used to generate transgenes are indicated with selectable marker denoted (*) [19]. Relative allele ratios of SNPs in ES lines carrying derivatives of BAC RP23-391D18 are represented as (129 + BAC)/CAST. Error bars indicate standard deviation of technical triplicates. Based on SNP ratios, red bar indicates transgene content in each line with the 3′ end positioned at the midpoint of the breakpoint interval. nd: not determined.

Previous studies focused on four full-length BAC transgenes that were derived from two overlapping BACs [19] (Figure 1). However, by PCR analysis of BAC-backbone sequences, six additional female ES lines carry X-linked integrants of the BAC RP23-391D18 with partial deletions. We turned to these truncated transgenes to further delimit sequences that dictate XCI states at the *Kdm5c* locus.

To determine transgene content and copy number, we exploited allele differences between the 129 and *M.m.* castaneous (CAST) X chromosomes in the ES cell line and assayed for the presence or absence of an additional BAC-transgene allele. Allele ratios for up to 18 SNPs across the region were measured using a quantitative primer-extension assay, qSNaPshot [13], [19], with primers that abut each SNP. The approach was validated with allele ratios of 2∶1 ((129+BAC)/CAST) for all SNPs mapping within full-length, single-copy BACs (e.g. B202) [19] (Figure 1, Figure S1). Similar analysis excluded three lines with multi-copy inserts (Figure S1). Further, the transgene in the B176 line is severely truncated and deletes the entire *Kdm5c-tg*.

Breakpoint analysis for two other transgene lines revealed deletions of distal XCI boundary sequences. ES lines C048 and C138 carry single-copy inserts that retain all or most of *Kdm5c-tg* (Figure 1). The C048 transgene contains the *AK148627* lncRNA but deletes a large portion of non-transcribed XCI boundary sequence. The transgene in C138 is more extensively deleted as all sequences downstream of *Kdm5c* are removed including the lncRNA. Additional SNPs narrowed the C138 transgene breakpoint to a small ∼900 bp window and indicate that at least 90% of the *Kdm5c* genomic locus remains intact. Further, by RNA fluorescence *in situ* hybridization (FISH) a stable nascent *Kdm5c-tg* transcript is detected in pre-XCI undifferentiated ES cells (not shown). Therefore, the C048 and C138 transgenes lack all or part of the intervening region between the 3′ end of escapee *Kdm5c* and the closest X-inactivated gene and allow the role of sequences within an escape domain and at an XCI boundary to be evaluated.

### Transgenes integrated into normally X-inactivated regions

Prior to examining transgene expression we surveyed the local chromosomal environment flanking the C048 and C138 BAC transgenes. By inverse PCR and subsequent analysis of an adjacent SNP, the C048 transgene inserted on the CAST X, upstream of the first coding exon of the *Mid1* gene (166,290,616 bp, mm9). Importantly, *Mid1* is normally X inactivated on the CAST X [19]. Additionally, FISH and SNP screening indicate that this transgene insertion was accompanied by a large and likely terminal deletion that removes the entire pseudoautosomal region (Figure S2A).

Similar characterization of the C138 transgene revealed that the BAC integrated on the CAST X at 98,065,555 bp (mm9) (Figure S2B). DNA FISH and SNP analysis near the C138 transgene integration site ensured that the BAC insertion was not accompanied by a larger chromosomal rearrangement or deletion (Figure S2B). This places *Kdm5c-tg* in an intron near the 3′ end of *Tex11*, a gene that functions in male meiosis [27], [28]. Although predominantly expressed in testis [29], we detected a low level of *Tex11* expression in somatic tissues by RT-PCR; monoallelic expression of a transcribed polymorphism in female fibroblasts with non-random XCI confirms that *Tex11* is normally X inactivated (Figure S2C). Therefore, both transgenes integrated into regions that are normally silenced by XCI, enabling direct testing of BAC sequence influences on *Kdm5c-tg* expression.

### Transgenic *Kdm5c* escapes XCI

**Figure 2:**
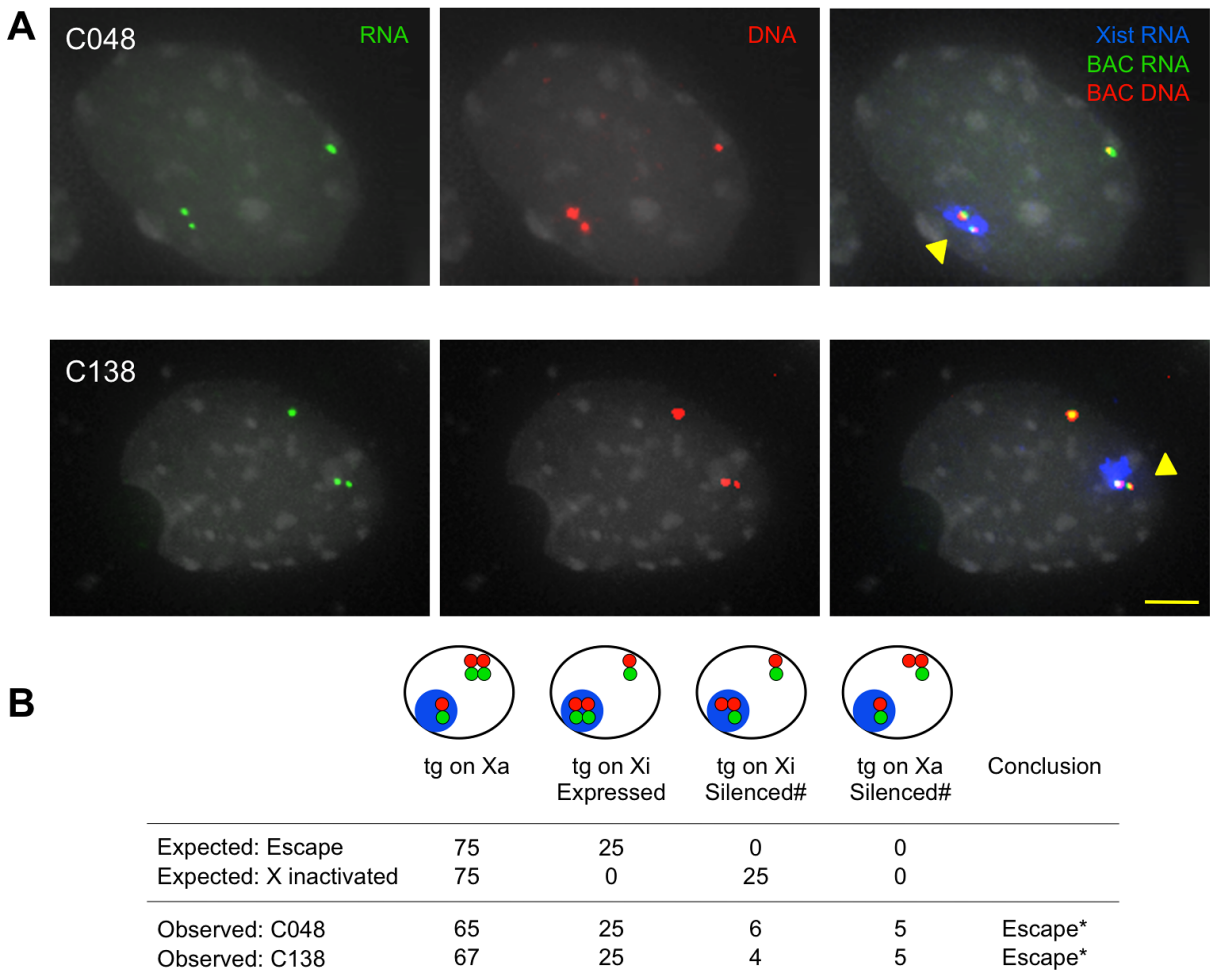
XCI status of *Kdm5c-tg* by sequential RNA and DNA FISH. (A) Representative nuclei showing *Kdm5c-tg* escapes XCI. *Kdm5c* BAC probes detect nascent transcripts (green) and DNA loci (red). *Xist* RNA (pseudocolored blue) identifies the inactive X (Xi). (B) Observed results were compared to expected values for the transgene to either escape or be X inactivated as influenced by XCI skewing [19]. Cartoons depict hybridization patterns scored, with colors as in A. (#) indicates that an absent transcript signal from the transgenic X (Xa or Xi) could reflect the endogenous or transgene locus, but cells were scored as silencing *Kdm5c-tg*. n = 100. *p<0.001.

Will *Kdm5c-tg* still escape XCI in the absence of distal boundary sequences? Expression was examined by sequential RNA and DNA FISH upon ES cell differentiation and concomitant XCI. Non-denatured cells were hybridized with a *Kdm5c* BAC probe to detect nascent transcripts from the endogenous and transgenic loci. Following probe fixation, cells were denatured and hybridized for DNA FISH to demarcate all *Kdm5c* loci. In C138 and C048, three expressed foci were detected in most cells (Figure 2). Importantly for each line, nuclei with two RNA signals colocalizing with *Xist* RNA demonstrate that both endogenous and transgenic loci are expressed on the inactive X. Additional FISH for C138 directly confirmed *Kdm5c-tg* escape, as one inactive X transcript colocalizes with a DNA signal from a probe at the integration site (Figure S3A). RNA FISH using a smaller *Kdm5c*-specific probe ensured results reflect *Kdm5c* expression (Figure S3B). Because of genetic background differences in the ES cells, XCI is skewed and the transgene is on the inactive CAST X in ∼25% of cells [19], [30]. For both C138 and C048, the proportion of cells with two expressed *Kdm5c* foci from the inactive X closely mirrors the frequency that cells inactive the CAST X chromosome (Figure 2B, Figure S3). Therefore, these data indicate *Kdm5c-tg* escapes XCI at a frequency similar to the non-transgenic locus.

To better estimate the level of *Kdm5c-tg* escape in C138, we isolated a clonal line that carries the transgene on the inactive X chromosome. Allelic expression, measured by qSNaPshot, is consistent with *Kdm5c-tg* and the non-transgenic locus each partially escaping XCI, at levels that are ∼34% of active X expression (see methods). Such levels are in good agreement with previous reports of partial escape for the endogenous locus [18], [19], [31], [32]. These data indicate that despite BAC truncation, *Kdm5c-tg* is expressed from the inactive X chromosome. Altogether, we conclude that *Kdm5c* escape does not require distal sequences.

### Transgene induces an active structural conformation

**Figure 3:**
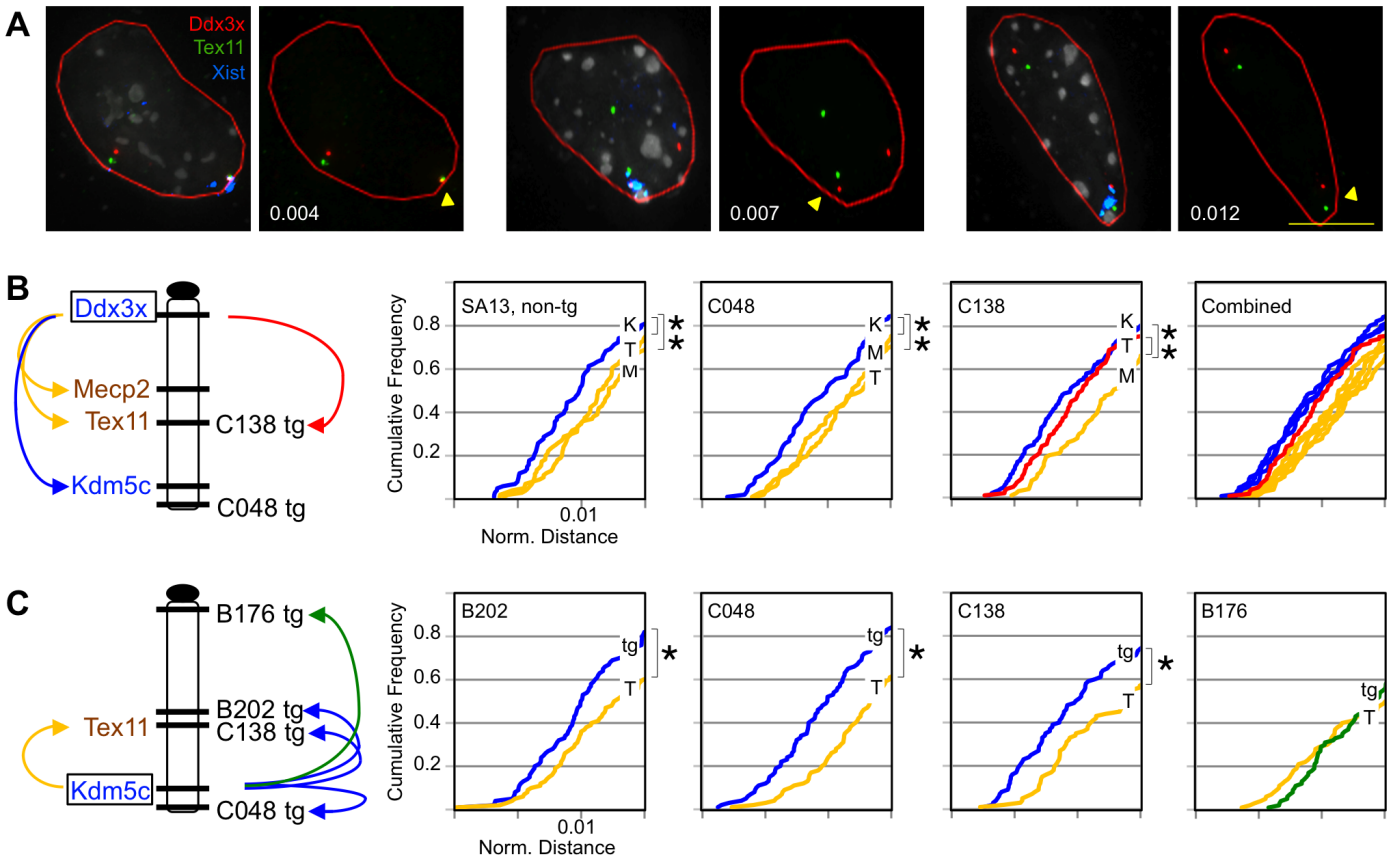
Transgenes assume frequent long-range escape-gene interactions. (A) Representative C138 nuclei compressed from 3D-image stacks hybridized to detect BAC DNA and *Xist* RNA. Arrowhead denotes inactive X. Normalized distances indicated (µm/µm^2^) reflect inactive X, 3D-probe distances (0.32, 0.89, and 1.33 µm) normalized to nuclear area (red outline). (B,C) Chromosome location of transgenes and BAC probes. BACs tested include X-inactivated genes *Mecp2* (M) and *Tex11* (T) (yellow/brown) or escapee *Kdm5c* (K) (blue). (B) Cumulative frequency curves show loci scored relative to *Ddx3x.* The shifted *Ddx3x*-*Tex11* curve in C138 (red) reflects transgene association in the subset of cells that inactivate the transgenic X. (C) Inactive X distances between *Kdm5c* and *Kdm5c-tg* (tg) are compared to endogenous *Kdm5c* and *Tex11* (T) on the non-transgenic X. B176 (green) lacks an expressed *Kdm5c-tg*. All plots include the 60–80% of cells with closest probe distances (complete data in Figure S5). Each probe comparison includes 100–150 nuclei. *p<0.02.

Previous studies of *Kdm5c* indicate that escape genes preferentially assume an exterior location on the *Xist*-coated inactive X in interphase nuclei [18]. This positioning likely facilitates more frequent long-range associations with other escape genes than with X-inactivated genes [17]. To further confirm the active state of *Kdm5c-tg*, we asked if transgenes establish similar interactions with distant escapees. Interactions were evaluated in differentiated post-XCI cells by FISH using three-dimensional deconvolution microscopy (Figure 3A). Inactive X distances were initially measured between the escapee *Ddx3x* and a probe detecting either escapee *Kdm5c* or an X-inactivated gene (Figure 3A,B). For each comparison, cumulative frequency plots indicate the proportion of nuclei in which two loci are closer than a given nuclear distance (normalized for area) (Figure 3B). This approach was first validated in a non-transgenic line and confirmed that profiles differ for the active and inactive X [17]; distant loci are more frequently in close proximity on the inactive X relative to their distance on the active X (Figure S4A). Further, inactive X escapee associations are also consistent with previous observations [17]. A higher proportion of nuclei have two escape loci in close proximity as the cumulative frequency plot of nuclear distances between escapees *Ddx3x* and *Kdm5c* is significantly shifted to the left relative to profiles comparing *Ddx3x* and either X-inactivated gene, *Tex11* or *Mecp2* (Figure 3B, Figure S5A). All differences were readily apparent regardless of whether or not probe distances were normalized to nuclear area (Figure S4B).

Similar probe comparisons were performed in the transgene lines. All profiles in line C048, with the *Mid1*-integrated transgene, were indistinguishable from the non-transgenic line (Figure 3B) indicating that a transgene at a location unrelated to the genes tested is insufficient to alter gene localization and interaction. In contrast, while C138 cumulative frequency curves comparing *Ddx3x* to active and inactive non-transgenic loci mirrored the other lines tested, comparison to the *Tex11* BAC revealed a significant left shift (Figure 3B, Figure S5A). *Tex11* lies at the C138 transgene integration site and proxies for the transgene in cells that inactivate the transgenic X. Indeed, the *Tex11* BAC is frequently located near *Ddx3x* on the inactive X, with a profile that is more similar to plots comparing two escapees than to curves for genes with differing XCI states, e.g. *Ddx3x* and *Mecp2*. These data suggest that a transgene can reconfigure associations on the inactive X.

To more directly visualize transgene interactions we specifically scored transgenic inactive X associations between *Kdm5c-tg* and the endogenous *Kdm5c* locus. Compared to interactions with X-inactivated *Tex11* (measured on non-transgenic inactive Xs), *Kdm5c* more frequently lies in close proximity to the transgene in C048, C138, and the full-length B202 transgene (Figure 3C, Figure S5B). In contrast, profiles for the severely truncated transgene in B176 resemble those with X-inactivated locus *Tex11* (Figure 3C, Figure S5B). Such a profile likely reflects the absence of *Kdm5c-tg* transcript in this line and indicates that the partial proximal boundary sequences retained in B176 are insufficient to direct interactions with escape loci. Importantly, these studies demonstrate that *Kdm5c-tg* in C138 and C048 structurally interacts in a manner similar to the endogenous locus, further confirming the active state of the transgenes on the inactive X. Therefore, despite truncating the endogenous escape domain, retained sequences are sufficient to induce an altered inactive X conformation even when inserted at a different chromosomal location.

### A role for distal boundary sequences in silencing adjacent genes

**Figure 4:**
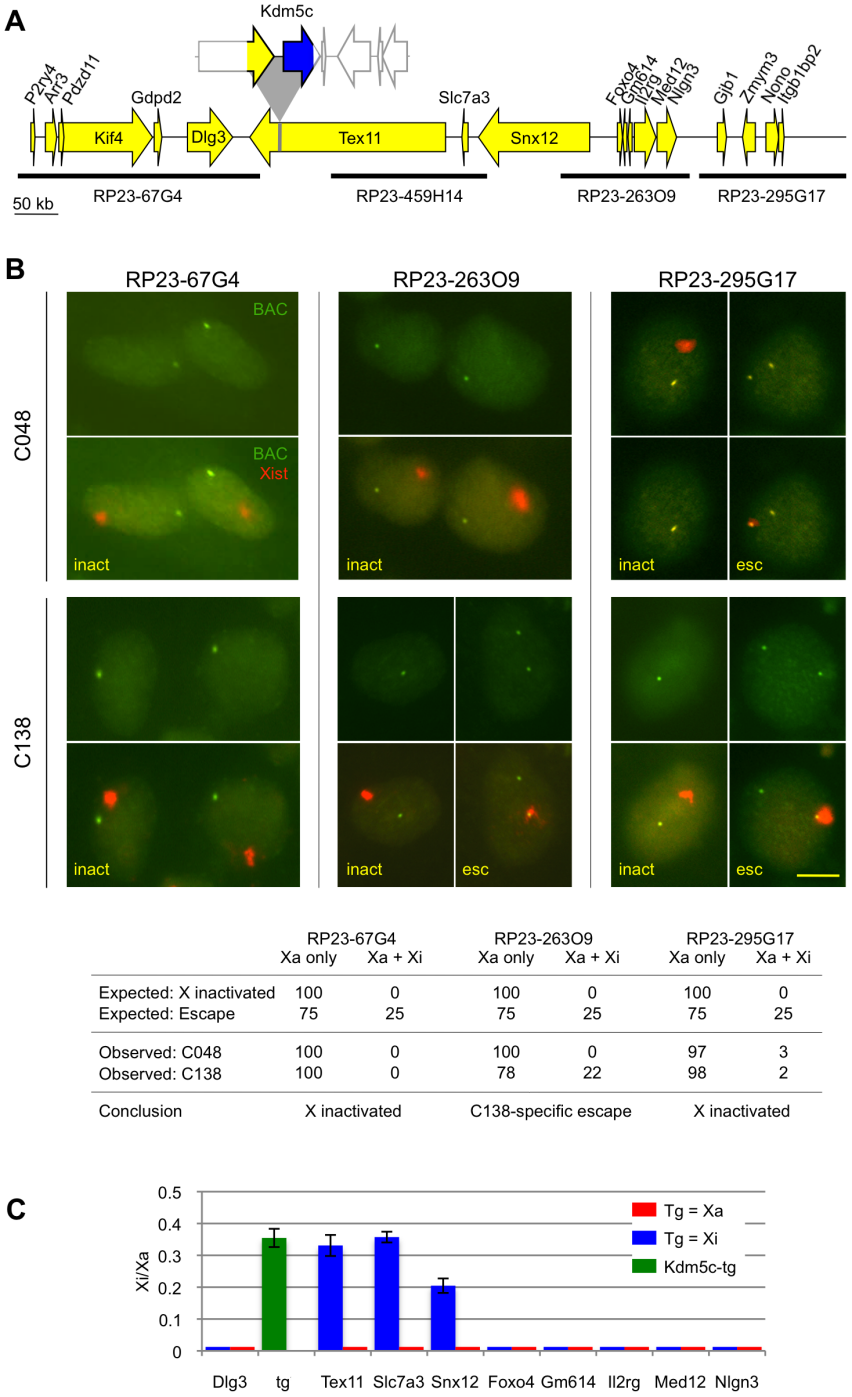
Disruption of distal XCI regulation. (A) *Tex11* locus with the C138 transgene drawn to scale. (B) RNA FISH for BAC probes flanking the C138 transgene insertion are compared to C048 (non-transgenic at the *Tex11* locus). Scale bar = 10 µm. Expected values used to conclude aberrant escape (p<0.001) consider the proportion of cells that inactivate the transgenic X due to XCI skewing. 100 cells were scored and the percentage with each expression pattern is indicated. (C) XCI status of clonal C138 cell lines that carry the transgene on the active or inactive X. Inactive X expression is indicated as a proportion of active X expression. *Kdm5c-tg* escape was estimated as described (see methods). No inactive X expression was detected for most genes tested, indicating that levels are at or below assay sensitivity of ∼1% of active X expression. All reactions were performed at least twice, with error bars indicating the standard deviation of technical triplicates.

We previously established that the full-length BAC transgenes retain intact XCI boundaries as *Kdm5c-tg* is expressed, but adjacent transgenic *Tspyl2* or *Iqsec2* properly undergo XCI [19]. Therefore, we next sought to determine if transcripts near the integration site would remain silent despite the absence of distal boundary sequences (Figure 4A). Given the orientation and close proximity of the C048 transgene to the pseudoautosomal boundary (Figure S2A) we focused on the C138 line. C138 proximal transgene sequences and XCI expression boundary are intact and therefore, adjacent genes are predicted to remain X inactivated. Consistent with this expectation, robust mono-allelic expression from the active X was detected by RNA FISH in both C048 (used to control for a non-transgenic *Tex11* locus) and C138 (Figure 4B). These data further establish that transcripts in this region are normally X inactivated and are not altered upon transgene integration.

To examine effects at the C138 distal boundary we queried transcripts included in BAC RP23-263O9 because low *Tex11* expression was undetectable on either X by RNA FISH (Figure S6). Monoallelic expression from only the active X in C048 confirms that RP23-263O9 transcripts are normally X inactivated (Figure 4B). However, a heterogeneous pattern was seen in C138, with inactive X expression in 22% of cells. This proportion closely approximates the percentage of cells that inactivate the transgenic CAST X (Figure 4B), and argues that distal genes on the transgenic X escape XCI at a high frequency. Aberrant XCI regulation does not extend further, as adjacent transcripts detected by BAC RP23-295G17 are properly X inactivated (Figure 4B).

To confirm and extend these results, we determined the XCI status of proximal and distal transcripts in differentiated clonal lines that carry the C138 transgene only on the active X or only on the inactive X chromosome. First, allele-specific expression of cDNA from the C138-derived clonal lines confirmed that the proximal gene *Dlg3* is X inactivated (Figure 4C). Next, *Tex11* at the integration site was tested. While *Tex11* is X inactivated in the clonal line that carries the transgene on the active X (Figure 4C), the gene now escapes XCI when interrupted by *Kdm5c-tg*. To determine the extent of XCI misregulation, we queried additional genes downstream of *Tex11*. Two additional transcripts, *Slc7a3* and *Snx12*, aberrantly escape XCI on the transgenic X (Figure 4C). By qSNaPshot, the level of inactive X escape relative to active X expression is quite similar for all three genes. However, it is unlikely that absolute inactive X expression is equivalent given that RNA FISH suggests significantly higher *Snx12* transcription on both Xs (Figure 4B, Figure S6). Altogether these results argue that absence of the distal XCI boundary results in 350 kb expansion of an escape domain.

## Discussion

Recent genome-wide studies have made tremendous strides in uncovering long-range organization and predicting functional domains [33]. Direct annotation of the inactive X is more challenging, in part because it is masked by its active X counterpart. Despite recent efforts to catalogue allele-specific epigenetic features (e.g. [10], [11], [34]), current understanding of the pivotal sequences and modifications that regulate how a gene responds to XCI remains incomplete. While inactive X profiling has identified intriguing candidates, functional dissection can reveal unexpected regulatory modes, such as uncovered here at *Kdm5c*.

These studies have expanded our understanding of the *Kdm5c* locus. Because our BAC transgenes carry large inserts encompassing X-chromosome genes that normally are influenced by XCI, effects are expected to recapitulate endogenous regulation and identify candidate sequences that are highly likely to be relevant. Our previous full-length BAC transgene studies allowed us to conclude that an element(s) within the BAC is sufficient to initiate *Kdm5c-tg* escape [19]. Such a regulatory element could also explain XCI escape of a human autosomal transgene [35]. For the *Kdm5c* locus, this activity was mapped to a 112 kb region defined by BAC overlap (Figure 1) [19]. Here we examine additional transgenes that further narrow this interval, as *Kdm5c-tg* still escapes XCI from BAC transgenes lacking distal boundary sequences (Figure 2). Because the truncated BACs integrated into X-inactivated regions, we conclude that the remaining transgene sequences must include a dominant element(s) sufficient to initiate *Kdm5c* escape and to structurally remodel the X in a manner that allows preferential association with escape genes (Figure 3). Further, our studies of the C138 transgene reveal an additional role for distal XCI boundary sequences, since in contrast to the full-length BACs [19] XCI regulation of adjacent X-inactivated genes was disrupted (Figure 4).

**Figure 5:**
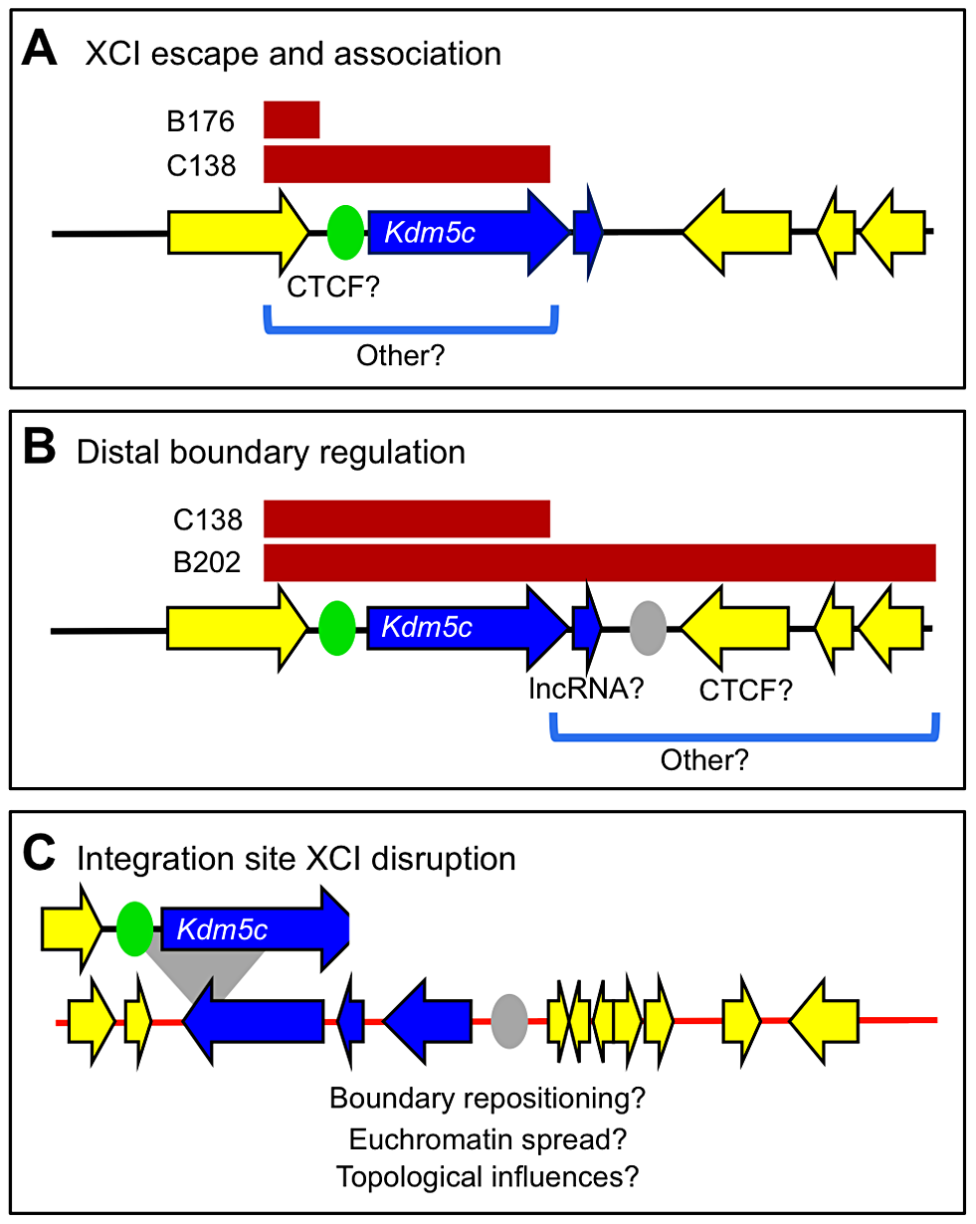
Two genetically separable regulatory activities at the *Kdm5c* locus. (A,B) Endogenous *Kdm5c* locus with transcripts ordered and XCI states annotated as in Figure 1 (not to scale). (A) An escape element that maps within the C138 BAC sequences (bracketed) is sufficient to direct *Kdm5c-tg* escape and long-distance escapee interactions. A previously described CTCF site is proposed to demarcate proximal boundary (green oval) [24]. (B) Regulation at the distal XCI boundary. While sequences sufficient to establish a distal XCI boundary (gray oval), are present in full length B202 BAC, they are absent in the short C138 transgene. (C) C138 transgene integration into the *Tex11* locus (not to scale) results in aberrant inactive X expression of three distal genes (blue).

What sequences are necessary for XCI escape and do these elements also facilitate long-range escapee interactions? Sequences orchestrating these activities must map within the C138 transgene and likely reside within the proximal XCI boundary (Figure 5A). Therefore, the complete escape domain, including the escapee lncRNA, cannot be necessary for directing inactive X expression. Retained BAC sequences include the *Kdm5c* promoter and CTCF-binding sites that are proposed to delimit this proximal XCI boundary [24] (Figure 5A). Nevertheless, CTCF binding alone is not sufficient to confer XCI escape [36]. Further, whether specific promoter elements alone can drive escape is untested, but large-scale transgenesis likely excludes promoter strength as a sole property [35]. Sequences within C138 also enable long-distance association with other escape genes. Yet, the region may be further narrowed as the short B176 transgene, lacking *Kdm5c-tg* and its promoter, fails to preferentially interact.

Deletion of distal transgene sequences in C138 reveals additional regulation at *Kdm5c*. In the absence of an XCI boundary, three normally X-inactivated genes near the BAC integration site now escape XCI (Figure 4). We asked whether aberrant distal expression is due to permissive chromatin propagated by read-though transcription from the truncated *Kdm5c-tg*. This possibility seems unlikely, as transcription does not extend across the entire escape domain (Figure S6). Further any read-through is at most minimal, as no transcription across the *Tex11* locus is seen by RNA FISH, even when the transgene is on the active X. Nevertheless, strand-specific RT-PCR within *Tex11* detects low-level sense and antisense transcripts from both non-transgenic and transgenic undifferentiated ES cells (Figure S6). That these transcripts are not unique to the *Kdm5c-tg* locus argues that low levels of transcription alone cannot enable escape. Therefore, while the extent that XCI is disrupted is likely dependent on integration site characteristics, the C138 transgene must lack a regulatory element that normally has an essential role in establishing an XCI boundary at the endogenous *Kdm5c* locus (Figure 5B). How this element functions is not clear, but could actively prevent heterochromatin encroachment into active domains or instead block escapee regulators from influencing adjacent silenced genes. Consistent with the former, a chromatin barrier could act as a boundary if upon deletion other distal elements reposition the XCI boundary (Figure 5C). CTCF could perform such a function, as sites are found near the distal *Kdm5c* boundary and are normally present at locations that could delimit the expanded escape domain (Figure S7). Moreover, CTCF frequently binds at chromatin boundaries throughout the genome [37], and can organize and reorganize chromatin loops [38]–[40]. This would suggest plasticity at XCI boundaries and could explain tissue differences in some escape genes [11], [17], [41].

Sequences at the distal XCI boundary could instead actively block adjacent genes from escape in a manner that is directional and in *cis* (Figure 5C). Deletion of such a boundary could appear as euchromatin spreading, although, to our knowledge, similar effects have not been described elsewhere in the genome. Yet, elements at other loci could explain this observation. CTCF functioning as an enhancer-blocking element fits this model [42]–[44], particularly since deletion at other epigenetically regulated loci can induce gene reactivation [45]. Alternatively, transcripts near escape genes may require additional elements to be properly X inactivated [46]. In this role, the lncRNA could silence by transcriptional interference [47], although effects extending such distances are not reported. Further, lncRNAs can recruit chromatin-modifying enzymes in *cis* (e.g. [47], [48]). Supporting recruitment, it is intriguing the *AK148627* lncRNA is amongst transcripts immunoprecipitated by the PRC2 polycomb-complex component EZH2 [49].

Finally, we considered the role that inactive X topological structure plays in determining XCI states. Distant escapee contacts are maintained for *Kdm5c-tg* at all three ectopic locations tested. Therefore, long-distance interaction is another inherent property of an escape locus, yet its mechanistic relationship to active transcription remains undefined. Transgenic loci are likely repositioned at the exterior of the *Xist* compartment, similar to endogenous *Kdm5c* [18]. Such rearrangement would also impact genes adjacent to the transgenes. While positioning on the inactive X could influence distal gene escape in C138, it cannot be sufficient since proximal genes remain X inactivated. Additional factors must be necessary to direct XCI fates.

Epigenomic features may refine the XCI boundary and localize key regulatory sequences. Using available data sets, H3K27me3 profiles in non-transgenic female lines mirror inactive X expression, with depletion clearly characterizing the expressed *Kdm5c* locus (Figure S7A). Intriguingly, while the proximal H3K27me3 transition is quite distinct, the distal boundary appears more diffuse (Figure S7A). Both H3K27me3 patterns occur at domain boundaries throughout the genome [50] and the distal profile may be indicative of an expression transition [9]. That this moderate H3K27me3 region contains critical regulatory sequences is supported by our current studies, since the shortest transgene breakpoint directly abuts this region. Nevertheless, the nature of the boundary makes regulatory element localization more difficult. If boundary repositioning expands the escape domain, it is intriguing that the novel boundary appears demarcated by H3K27me3 even on non-transgenic chromosomes (Figure S7B). However, further conclusions will require chromatin profiling on transgenic chromosomes. We next turned to DNaseI hypersensitivity that demarcates many regulatory elements [51]. At both the endogenous *Kdm5c* locus and C138 transgene integration site available data only identify hypersensitive sites at gene promoters and CTCF-binding sites (Figure S7). Perhaps this strengthens CTCF as a candidate. A caveat is that such a function may be developmentally regulated and no female lines have been profiled upon the onset of XCI.

Altogether, work here has defined two separable functions at the *Kdm5c* locus. We narrowed sequences required for directing escape and for the first time have assigned a function to an XCI boundary in actively delimiting expression domains. By defining and demarcating regions responsible for each activity, future experiments can be directed to examine specific candidate elements.

## Methods

### Transgenic cell lines

The parental ES line SA13 was derived from a (129×CAST)F1 female [19]. ES cell lines carrying X-linked BAC transgene RP23-391D18 were described previously [19]. All cells were cultured using established conditions and were maintained in the absence of drug selection [19]. For post-XCI experiments, cells were differentiated for ten days following LIF removal.

Clonal C138 lines were isolated by first differentiating ES lines for 10 days. Cells were replated using conditions that further enrich for differentiated cells [19] and after two days were infected with SV40-VA4554 [52]. Cells were passaged as required and after >20 days plated at very low cell density and allowed to clonally expand. Monoallelic expression of SNPs within *Hprt* and/or *Pctk1* [32] confirmed clonality.

### Mapping transgene breakpoints

Due to the location of the selectable marker within the RP23-391D18 BAC vector [19], truncated transgenic lines surviving initial drug selection lack genomic sequences at the distal XCI boundary. Informative SNPs to delimit these transgene breakpoints were identified (http://cgd.jax.org/cgdsnpdb) and are listed in Table S1. Allelic ratios were evaluated using a quantitative primer extension assay, qSNaPshot [13], [19]. Samples were run on an ABI 3130XL sequencer and peak heights measured using GeneMapper 4.0 software with SNaPshot default settings. Allele ratios in transgenic lines were normalized by comparison with the non-transgenic ES line. Results were further adjusted as allele ratios for a non-transgenic SNP rs29296320 deviated slightly from an expected ratio of 1.0 (ranging from 0.84 to 1.07), likely reflecting loss of an X in a small proportion of cells.

### Identification of transgene integration sites

Precise transgene integration sites were determined by inverse PCR [53]. For C048 and C138, genomic DNA was digested with XbaI or PstI respectively. Purified DNA was self-ligated in dilute conditions and used as template for PCR with BAC-derived primers. PCR products were cloned and sequenced. Similar efforts for B176 failed to isolate integration sequences, consistent with a more complex vector rearrangement upon insertion. To determine if C138 and C048 transgene integrations resulted in large-scale deletions, genomic SNPs distal to the integration site were analyzed by qSNAPshot (Table S1 for primers and SNPs).

To identify the strain origin of the transgenic Xs in C138 and C048, SNP alleles were assayed from transgenic X specific PCR products that were generated by anchoring one primer to the BAC backbone. For C048, the closest informative SNP was >6 kb away and required initial amplification from a self-ligated template, similar to inverse PCR (Table S1 for SNP and primer information). Strain origin of the transgenic X in additional lines was inferred by determining the frequency that the BAC is on the inactive X since XCI skewing results in inactivation of the CAST X in 25% of cells [19].

### Inactive X expression analysis

The normal XCI status of transcripts at the transgene integration site was assayed using qSNaPshot to measure allelic expression in the non-randomly X-inactivated mouse fibroblast lines B120 or B119 [13], [14]. *Mid1* was tested previously in a similar manner [19]. *Mid1* has a unique gene organization and XCI pattern; it straddles the pseudoautosomal (PAR) boundary in some strains, but is X-specific in others [54], [55] (Figure S2A). *Mid1* escapes XCI in domestic mouse strains [10], [54], but is X inactivated on the CAST X [19].

Allelic expression was similarly assayed in the C138 clonal lines. For genes flanking the transgene, inactive X expression was measured relative to the active X allele and normalized to DNA. *Kdm5c*, with three expressed loci in C138, required the expression ratio to be normalized to non-transgenic DNA (to account for dye incorporation differences) and additionally to DNA from the clonal line (to account for loss of an X in a small subset of cells). However, both *Kdm5c-tg* and the endogenous locus on the active X are derived from domestic strains and are not distinguishable. Therefore, levels of *Kdm5c-tg* escape were estimated from the normalized allele ratios as if equivalent to the endogenous CAST inactive X allele. This estimate appears justified since both inactive X alleles (CAST and *Kdm5c-tg*) are predicted to partially escape at levels similar to those previously reported [18], [19], [31], [32]. Further, given the measured allele ratios, estimates of lower *Kdm5c-tg* escape require concomitant reduction in the endogenous CAST allele to levels below that been previously seen.

### Fluorescent in situ hybridization

FISH probes included *Xist* (7.2 kb of exon 1) [19], *Kdm5c* (19 kb spanning exons 5–12 [19]), DXWas70, an X-specific repeat [56], and BACs RP23-391D18 (includes *Kdm5c*), RP23-330G24 (*Kdm5c*), RP23-67G4, RP23-459H14 (Tex11), RP23-263O9, RP24-255O24, RP23-295G17, RP23-459P19 (*Ddx3x*), and RP23-378I14 (*Mecp2*). Probes were directly labeled with Alexa Fluors 488, 594, or 647 by nick translation using either ARES DNA labeling kits (Invitrogen) or ChromaTide Alexa Fluor dUTPs (Invitrogen) as indicated by the manufacturer.

Slides were prepared and FISH performed for each specific experiment as follows. For DNA FISH studies, metaphase spreads were prepared and FISH performed as previously described [19], [57]. For all other studies, embryoid bodies were plated on slides at day 3 of differentiation and cultured to day 10. RNA FISH was performed on non-denatured slides as described [18], [58]. For sequential RNA and DNA FISH, slides were initially processed as for RNA FISH. Subsequently, signals were fixed in 4% paraformaldehyde in PBS prior to denaturing (75°C for 5 minutes) and processing for DNA FISH [19]. For association studies, cells were fixed in 4% paraformaldehyde before permeablization to preserve nuclear morphology [17]. Slides were denatured at 85°C for 4′ or 75°C for 7′, which allowed sufficient retention of *Xist* RNA to identify the inactive X chromosome.

Slides were imaged on Nikon TE2000-U microscope with Roper Scientific CCD camera and NIS elements software equipped with a 60× objective. Alternatively, a DeltaVision Elite microscope was used that is equipped with 60× or 100× objective and CoolSnap HQ2 Photometrics camera. Deltavision images were acquired across 0.2 µm Z stacks, deconvolved, and analyzed using softWoRx software version 5.5.5. In all cases, wavelengths were captured separately and merged and pseudocolored in Adobe Photoshop. Image manipulation was restricted to overall fluorescent level adjustment applied uniformly across the image.

### RNA FISH analysis

To ensure optimal hybridization, we adopted specific scoring criteria for each experiment. For all FISH expression studies, we required hybridization patterns for scored cells to at least reflect known endogenous XCI expression. That is, for a gene that normally escapes XCI (*Kdm5c*), all cells included had at least one active X and one inactive X signal; for normally X-inactivated genes, only cells with at least one robust active X signal were scored. Additional RNA signals then reflect transgene expression (*Kdm5c* escape) or aberrant escape (for normally X-inactivated genes). Multiple planes were examined to ensure that out-of-focus signals were not excluded. Assignment of *Kdm5c* signals was facilitated by colocalization with BAC DNA FISH signals to pinpoint the *Kdm5c* locus or the integration site locus. Unless noted, each experiment scored at least 100 nuclei that met criteria, with each scored cell selected from an independent field of vision. Statistical significance was evaluated by Chi square analysis.

### Structural association analysis

To evaluate probe association slides were viewed on a DeltaVision Elite microscope (100× objective). X,Y,Z coordinates were recorded for each signal and the 3D distance between probes calculated [17]. Nuclear area was calculated by averaging polygon areas (demarcating the nucleus) across all in focus Z sections and was used to normalize for differences in nuclear size and morphology. Analysis was limited to ∼95% of cells with nuclear area <175 µm^2^ to ensure overlapping distributions across all cell lines. For each probe set 100–150 nuclei were scored per cell line. Significance was assessed using a Kolmogorov-Smirnov two-sample statistic [59].

## Supporting Information

Figure S1 Characterization of additional RP23-391D18-derived transgenes. Transgenes were evaluated and annotated as in Figure 1. Based on SNP ratios, red bar indicates transgene content in each line with the 3′ end drawn at the midpoint of the breakpoint interval. Double bars indicate an increase in transgene copy number. nd: not determined.
(TIF)

Figure S2 Characterization of transgene integration sites. (A) C048 transgene insertion within the *Mid1* gene at Xqter is correctly oriented and to scale, although the transgene inserted on the CAST X chromosome. Available annotated sequence is derived from C57BL/6J (sequence gaps in gray (mm10)). *Mid1* spans the pseudoautosomal region (PAR) boundary (dotted line, [55]) in domestic mice, but is X specific in other mouse strains [54]. CAST PAR is distal, but imprecisely defined (green arrow). SNPs indicated by asterisks are deleted on the transgenic CAST X. To identify PAR BACs we screened GenBank high-throughput genomic sequences (HTGS) for clones that partially overlap annotated X and Y sequences. PAR localization of RP24-255O24 was established by FISH with hybridization to both CAST and domestic X chromosomes and to the Y chromosome. Subsequently, RP24-255O24 FISH confirmed PAR deletion on the transgenic C048 X. (B) Gene organization at the C138 integration site drawn to scale. Genomic SNPs (*) as close as 6 kb from the integration site are intact. By FISH, BACs that flank the integration site and the transgene BAC RP23-391D18 are colocalized and rule out large chromosomal alterations. (C) *Tex11* is normally X inactivated. Allelic expression of Tex11 SNP rs29083830 (red* in B) was examined in fibroblast line B120 that carries a CAST inactive X. Monoallelic expression from the *M.m*. domesticus (DOM) active X indicates *Tex11* is normally X inactivated.
(TIF)

Figure S3 *Kdm5c-tg* escapes XCI. (A) Sequential RNA and DNA FISH directly demonstrates *Kdm5c-tg* inactive X expression in C138 by colocalization with an integration site probe. Nuclei were hybridized to detect *Xist* RNA and *Kdm5c* RNA (BAC probe) and subsequently denatured and probed for DNA at the integration site (*Tex11* BAC). Cartoons represent FISH patterns scored. Results were compared to values expected for *Kdm5c-tg* to either escape or be X inactivated, as influenced by XCI skewing [19]. n = 50, with cells scored from 37 fields of vision. (B) RNA FISH using a *Kdm5c*-specific probe establishes *Kdm5c-tg* escapes XCI. Cells with patterns depicted in cartoons were scored as above. n = 100. Asterisk indicates cells with expression from only a single inactive X allele. We conservatively scored these cells as inactivating *Kdm5c-tg*, although the inactivated locus could be on either X and derived from either the transgenic or endogenous locus. Both C048 and C138 transgenes escape XCI with results mirroring those in Figure 2.
(TIF)

Figure S4 Long-range X-chromosome associations. (A) *Ddx3x* interactions with distant genes *Kdm5c*, *Tex11*, and *Mecp2* (as in Figure 3B) differ between active (black) and inactive (green) X chromosomes. Results are shown for the non-transgenic line SA13. (B,C) Interactions with active and inactive loci are apparent regardless of whether distances are normalized to nuclear area. Cumulative frequency plots with non-normalized distances for probe associations with (B) *Ddx3x* or (C) *Kdm5c* are shown for C138 or for all comparisons included in the combined plots from Figure 3B and Figure 3C. Colors are as in Figure 3B,C. *p<0.006.
(TIF)

Figure S5 Distribution of complete X-chromosome interaction distances. For all cells scored, normalized distances were binned for probes relative to (A) *Ddx3x* or (B) *Kdm5c*. Inverted triangles demarcate average normalized distance. Distributions shifted to the left indicate a larger number of nuclei with probes in close proximity. *p<0.02.
(TIF)

Figure S6 Transcriptional influences at the C138 integration site. (A) *Tex11* locus to demarcate FISH probe and RT-PCR amplicons tested. (B) Despite truncation of *Kdm5c-tg*, read-through transcripts extending across *Tex11* are not detected by RNA FISH. Double-stranded BAC probes used for FISH will detect both sense and antisense transcripts. Although robust *Kdm5c-tg* expression is apparent (first panel, labeled with Alexa594 and pseudocolored green), RNA FISH fails to detect sense (*Tex11*) or antisense expression (read-through from *Kdm5c-tg*) using BAC RP23-459H14 (middle panel). DNA FISH is included to demonstrate that the BACs are appropriately labeled. (C) Transcription downstream of *Kdm5c-tg* appears similar to non-transgenic loci. Strand-specific RT-PCR was performed using cDNA synthesized by reverse transcription with a specific primer. cDNAs were amplified for 35 cycles and low levels of *Tex11* (sense (s)) and antisense (as) transcripts were detected (at locus 1 in A) in undifferentiated C138 and non-transgenic (SA13) ES lines. (D) RT-PCR establishes that sequences downstream of *Tex11* are not expressed from transgenic or non-transgenic loci (locus 2 in A).
(TIF)

Figure S7 Genomic influences on escape-gene expression. (A) *Kdm5c* locus with C138 BAC indicated and escapee region shaded. (B) *Tex11* locus denoting the C138 integration site (inverted triangle) and aberrant-escape domain (shaded). Genomic coordinates at top are in Mb (mm9). Triangles mark CTCF sites that are conserved in the majority of available data sets (genome.ucsc.edu) (black) or as reported (gray) [24]. DNaseI hypersensitivity is shown for adult female GSM1014171. H3K27me3 occupancy tracks from GSM517917, GSM517918, and GSM905446 are displayed as described in the corresponding references [10], [60].
(TIF)

Table S1 Oligonucleotide primers. (*) Primer abuts SNP for qSNaPshot. Additional abbreviations: ^1^Primer amplifies transgenic X, ^2^Primer amplifies non-transgenic X, ^3^transgene-specific inverse PCR primer.
(PDF)

## References

[1] Dixon JR, Selvaraj S, Yue F, Kim A, Li Y, et al (2012) Topological domains in mammalian genomes identified by analysis of chromatin interactions. Nature 485: 376–380. 22495300 10.1038/nature11082 PMC3356448

[2] Ryba T, Hiratani I, Lu J, Itoh M, Kulik M, et al (2010) Evolutionarily conserved replication timing profiles predict long-range chromatin interactions and distinguish closely related cell types. Genome Res 20: 761–770. 20430782 10.1101/gr.099655.109 PMC2877573

[3] Lieberman-Aiden E, van Berkum NL, Williams L, Imakaev M, Ragoczy T, et al (2009) Comprehensive mapping of long-range interactions reveals folding principles of the human genome. Science 326: 289–293. 19815776 10.1126/science.1181369 PMC2858594

[4] Guelen L, Pagie L, Brasset E, Meuleman W, Faza MB, et al (2008) Domain organization of human chromosomes revealed by mapping of nuclear lamina interactions. Nature 453: 948–951. 18463634 10.1038/nature06947

[5] Wen B, Wu H, Shinkai Y, Irizarry RA, Feinberg AP (2009) Large histone H3 lysine 9 dimethylated chromatin blocks distinguish differentiated from embryonic stem cells. Nat Genet 41: 246–250. 19151716 10.1038/ng.297 PMC2632725

[6] Pauler FM, Sloane MA, Huang R, Regha K, Koerner MV, et al (2009) H3K27me3 forms BLOCs over silent genes and intergenic regions and specifies a histone banding pattern on a mouse autosomal chromosome. Genome Res 19: 221–233. 19047520 10.1101/gr.080861.108 PMC2652204

[7] Nora EP, Lajoie BR, Schulz EG, Giorgetti L, Okamoto I, et al (2012) Spatial partitioning of the regulatory landscape of the X-inactivation centre. Nature 485: 381–385. 22495304 10.1038/nature11049 PMC3555144

[8] Wutz A (2011) Gene silencing in X-chromosome inactivation: advances in understanding facultative heterochromatin formation. Nat Rev Genet 12: 542–553. 21765457 10.1038/nrg3035

[9] Berletch JB, Yang F, Xu J, Carrel L, Disteche CM (2011) Genes that escape from X inactivation. Hum Genet 130: 237–245. 21614513 10.1007/s00439-011-1011-z PMC3136209

[10] Pinter SF, Sadreyev RI, Yildirim E, Jeon Y, Ohsumi TK, et al (2012) Spreading of X chromosome inactivation via a hierarchy of defined Polycomb stations. Genome Res 22: 1864–76. 22948768 10.1101/gr.133751.111 PMC3460182

[11] Calabrese JM, Sun W, Song L, Mugford JW, Williams L, et al (2012) Site-specific silencing of regulatory elements as a mechanism of X inactivation. Cell 151: 951–963. 23178118 10.1016/j.cell.2012.10.037 PMC3511858

[12] Engreitz JM, Pandya-Jones A, McDonel P, Shishkin A, Sirokman K, et al (2013) The Xist lncRNA Exploits Three-Dimensional Genome Architecture to Spread Across the X Chromosome. Science 341: 1237973. 23828888 10.1126/science.1237973 PMC3778663

[13] Carrel L, Willard HF (2005) X-inactivation profile reveals extensive variability in X-linked gene expression in females. Nature 434: 400–404. 15772666 10.1038/nature03479

[14] Lopes AM, Arnold-Croop SE, Amorim A, Carrel L (2011) Clustered transcripts that escape X inactivation at mouse XqD. Mamm Genome 22: 572–582. 21769671 10.1007/s00335-011-9350-6

[15] Carrel L, Park C, Tyekucheva S, Dunn J, Chiaromonte F, et al (2006) Genomic environment predicts expression patterns on the human inactive X chromosome. PLoS Genet 2: e151. 17009873 10.1371/journal.pgen.0020151 PMC1584270

[16] Tsuchiya KD, Greally JM, Yi Y, Noel KP, Truong JP, et al (2004) Comparative sequence and X-inactivation analyses of a domain of escape in human Xp11.2 and the conserved segment in mouse. Genome Res 14: 1275–1284. 15197169 10.1101/gr.2575904 PMC442142

[17] Splinter E, de Wit E, Nora EP, Klous P, van de Werken HJ, et al (2011) The inactive X chromosome adopts a unique three-dimensional conformation that is dependent on Xist RNA. Genes Dev 25: 1371–1383. 21690198 10.1101/gad.633311 PMC3134081

[18] Chaumeil J, Le Baccon P, Wutz A, Heard E (2006) A novel role for Xist RNA in the formation of a repressive nuclear compartment into which genes are recruited when silenced. Genes Dev 20: 2223–2237. 16912274 10.1101/gad.380906 PMC1553206

[19] Li N, Carrel L (2008) Escape from X chromosome inactivation is an intrinsic property of the Jarid1c locus. Proc Natl Acad Sci U S A 105: 17055–17060. 18971342 10.1073/pnas.0807765105 PMC2579377

[20] Agulnik AI, Mitchell MJ, Mattei MG, Borsani G, Avner PA, et al (1994) A novel X gene with a widely transcribed Y-linked homologue escapes X-inactivation in mouse and human. Hum Mol Genet 3: 879–884. 7951230 10.1093/hmg/3.6.879

[21] Iwase S, Lan F, Bayliss P, de la Torre-Ubieta L, Huarte M, et al (2007) The X-linked mental retardation gene SMCX/JARID1C defines a family of histone H3 lysine 4 demethylases. Cell 128: 1077–1088. 17320160 10.1016/j.cell.2007.02.017

[22] Reinius B, Shi C, Hengshuo L, Sandhu KS, Radomska KJ, et al (2010) Female-biased expression of long non-coding RNAs in domains that escape X-inactivation in mouse. BMC Genomics 11: 614. 21047393 10.1186/1471-2164-11-614 PMC3091755

[23] Tsuchiya KD, Willard HF (2000) Chromosomal domains and escape from X inactivation: comparative X inactivation analysis in mouse and human. Mamm Genome 11: 849–854. 11003698 10.1007/s003350010175

[24] Filippova GN, Cheng MK, Moore JM, Truong JP, Hu YJ, et al (2005) Boundaries between chromosomal domains of X inactivation and escape bind CTCF and lack CpG methylation during early development. Dev Cell 8: 31–42. 15669143 10.1016/j.devcel.2004.10.018

[25] Phillips JE, Corces VG (2009) CTCF: master weaver of the genome. Cell 137: 1194–1211. 19563753 10.1016/j.cell.2009.06.001 PMC3040116

[26] Lee JT (2012) Epigenetic regulation by long noncoding RNAs. Science 338: 1435–1439. 23239728 10.1126/science.1231776

[27] Yang F, Gell K, van der Heijden GW, Eckardt S, Leu NA, et al (2008) Meiotic failure in male mice lacking an X-linked factor. Genes Dev 22: 682–691. 18316482 10.1101/gad.1613608 PMC2259036

[28] Adelman CA, Petrini JH (2008) ZIP4H (TEX11) deficiency in the mouse impairs meiotic double strand break repair and the regulation of crossing over. PLoS Genet 4: e1000042. 18369460 10.1371/journal.pgen.1000042 PMC2267488

[29] Wang PJ, McCarrey JR, Yang F, Page DC (2001) An abundance of X-linked genes expressed in spermatogonia. Nat Genet 27: 422–426. 11279525 10.1038/86927

[30] Lee JT, Lu N (1999) Targeted mutagenesis of Tsix leads to nonrandom X inactivation. Cell 99: 47–57. 10520993 10.1016/s0092-8674(00)80061-6

[31] Sheardown S, Norris D, Fisher A, Brockdorff N (1996) The mouse Smcx gene exhibits developmental and tissue specific variation in degree of escape from X inactivation. Hum Mol Genet 5: 1355–1360. 8872477 10.1093/hmg/5.9.1355

[32] Carrel L, Hunt PA, Willard HF (1996) Tissue and lineage-specific variation in inactive X chromosome expression of the murine Smcx gene. Hum Mol Genet 5: 1361–1366. 8872478 10.1093/hmg/5.9.1361

[33] Dunham I, Kundaje A, Aldred SF, Collins PJ, Davis CA, et al (2012) An integrated encyclopedia of DNA elements in the human genome. Nature 489: 57–74. 22955616 10.1038/nature11247 PMC3439153

[34] Sadreyev RI, Yildirim E, Pinter SF, Lee JT (2013) Bimodal quantitative relationships between histone modifications for X-linked and autosomal loci. Proc Natl Acad Sci U S A 110: 6949–6954. 23564346 10.1073/pnas.1216449110 PMC3637770

[35] Yang C, McLeod AJ, Cotton AM, de Leeuw CN, Laprise S, et al (2012) Targeting of over 1.5 Mb of human DNA into the mouse X chromosome reveals presence of cis-acting regulators of epigenetic silencing. Genetics 192: 1281–1293. 23023002 10.1534/genetics.112.143743 PMC3512139

[36] Ciavatta D, Kalantry S, Magnuson T, Smithies O (2006) A DNA insulator prevents repression of a targeted X-linked transgene but not its random or imprinted X inactivation. Proc Natl Acad Sci U S A 103: 9958–9963. 16777957 10.1073/pnas.0603754103 PMC1479543

[37] Cuddapah S, Jothi R, Schones DE, Roh TY, Cui K, et al (2009) Global analysis of the insulator binding protein CTCF in chromatin barrier regions reveals demarcation of active and repressive domains. Genome Res 19: 24–32. 19056695 10.1101/gr.082800.108 PMC2612964

[38] Yusufzai TM, Tagami H, Nakatani Y, Felsenfeld G (2004) CTCF tethers an insulator to subnuclear sites, suggesting shared insulator mechanisms across species. Mol Cell 13: 291–298. 14759373 10.1016/s1097-2765(04)00029-2

[39] Hou C, Zhao H, Tanimoto K, Dean A (2008) CTCF-dependent enhancer-blocking by alternative chromatin loop formation. Proc Natl Acad Sci U S A 105: 20398–20403. 19074263 10.1073/pnas.0808506106 PMC2629272

[40] Murrell A, Heeson S, Reik W (2004) Interaction between differentially methylated regions partitions the imprinted genes Igf2 and H19 into parent-specific chromatin loops. Nat Genet 36: 889–893. 15273689 10.1038/ng1402

[41] Reinius B, Johansson MM, Radomska KJ, Morrow EH, Pandey GK, et al (2012) Abundance of female-biased and paucity of male-biased somatically expressed genes on the mouse X-chromosome. BMC Genomics 13: 607. 23140559 10.1186/1471-2164-13-607 PMC3534601

[42] Bell AC, West AG, Felsenfeld G (1999) The protein CTCF is required for the enhancer blocking activity of vertebrate insulators. Cell 98: 387–396. 10458613 10.1016/s0092-8674(00)81967-4

[43] Saitoh N, Bell AC, Recillas-Targa F, West AG, Simpson M, et al (2000) Structural and functional conservation at the boundaries of the chicken beta-globin domain. EMBO J 19: 2315–2322. 10811622 10.1093/emboj/19.10.2315 PMC384375

[44] Hark AT, Schoenherr CJ, Katz DJ, Ingram RS, Levorse JM, et al (2000) CTCF mediates methylation-sensitive enhancer-blocking activity at the H19/Igf2 locus. Nature 405: 486–489. 10839547 10.1038/35013106

[45] Brenton JD, Drewell RA, Viville S, Hilton KJ, Barton SC, et al (1999) A silencer element identified in Drosophila is required for imprinting of H19 reporter transgenes in mice. Proc Natl Acad Sci U S A 96: 9242–9247. 10430927 10.1073/pnas.96.16.9242 PMC17764

[46] Chow JC, Ciaudo C, Fazzari MJ, Mise N, Servant N, et al (2010) LINE-1 activity in facultative heterochromatin formation during X chromosome inactivation. Cell 141: 956–969. 20550932 10.1016/j.cell.2010.04.042

[47] Nagano T, Mitchell JA, Sanz LA, Pauler FM, Ferguson-Smith AC, et al (2008) The Air noncoding RNA epigenetically silences transcription by targeting G9a to chromatin. Science 322: 1717–1720. 18988810 10.1126/science.1163802

[48] Pandey RR, Mondal T, Mohammad F, Enroth S, Redrup L, et al (2008) Kcnq1ot1 antisense noncoding RNA mediates lineage-specific transcriptional silencing through chromatin-level regulation. Mol Cell 32: 232–246. 18951091 10.1016/j.molcel.2008.08.022

[49] Zhao J, Ohsumi TK, Kung JT, Ogawa Y, Grau DJ, et al (2010) Genome-wide identification of polycomb-associated RNAs by RIP-seq. Mol Cell 40: 939–953. 21172659 10.1016/j.molcel.2010.12.011 PMC3021903

[50] Barkess G, West AG (2012) Chromatin insulator elements: establishing barriers to set heterochromatin boundaries. Epigenomics 4: 67–80. 22332659 10.2217/epi.11.112

[51] Song L, Zhang Z, Grasfeder LL, Boyle AP, Giresi PG, et al (2011) Open chromatin defined by DNaseI and FAIRE identifies regulatory elements that shape cell-type identity. Genome Res 21: 1757–1767. 21750106 10.1101/gr.121541.111 PMC3202292

[52] Tevethia MJ, Ozer HL (2001) SV40-mediated immortalization. Methods Mol Biol 165: 185–199. 11217385 10.1385/1-59259-117-5:185

[53] Li ZH, Liu DP, Liang CC (1999) Modified inverse PCR method for cloning the flanking sequences from human cell pools. Biotechniques 27: 660–662. 10524300 10.2144/99274bm05

[54] Dal Zotto L, Quaderi NA, Elliott R, Lingerfelter PA, Carrel L, et al (1998) The mouse Mid1 gene: implications for the pathogenesis of Opitz syndrome and the evolution of the mammalian pseudoautosomal region. Hum Mol Genet 7: 489–499. 9467009 10.1093/hmg/7.3.489

[55] Perry J, Palmer S, Gabriel A, Ashworth A (2001) A short pseudoautosomal region in laboratory mice. Genome Res 11: 1826–1832. 11691846 10.1101/gr.203001 PMC311143

[56] Disteche CM, Gandy SL, Adler DA (1987) Translocation and amplification of an X-chromosome DNA repeat in inbred strains of mice. Nucleic Acids Res 15: 4393–4401. 3588301 10.1093/nar/15.11.4393 PMC340869

[57] Miller AP, Gustashaw K, Wolff DJ, Rider SH, Monaco AP, et al (1995) Three genes that escape X chromosome inactivation are clustered within a 6 Mb YAC contig and STS map in Xp11.21-p11.22. Hum Mol Genet 4: 731–739. 7633424 10.1093/hmg/4.4.731

[58] Chaumeil J, Augui S, Chow JC, Heard E (2008) Combined immunofluorescence, RNA fluorescent in situ hybridization, and DNA fluorescent in situ hybridization to study chromatin changes, transcriptional activity, nuclear organization, and X-chromosome inactivation. Methods Mol Biol 463: 297–308. 18951174 10.1007/978-1-59745-406-3_18

[59] Xu N, Tsai CL, Lee JT (2006) Transient homologous chromosome pairing marks the onset of X inactivation. Science 311: 1149–1152. 16424298 10.1126/science.1122984

[60] Yang F, Babak T, Shendure J, Disteche CM (2010) Global survey of escape from X inactivation by RNA-sequencing in mouse. Genome Res 20: 614–622. 20363980 10.1101/gr.103200.109 PMC2860163

