# Supplementary figures and images for: Deletion of an X-Inactivation Boundary Disrupts Adjacent Gene Silencing
Source: PLoS Genet. 2013 Nov 21;9(11):e1003952. doi: 10.1371/journal.pgen.1003952 (PMC3836711; doi:10.1371/journal.pgen.1003952)

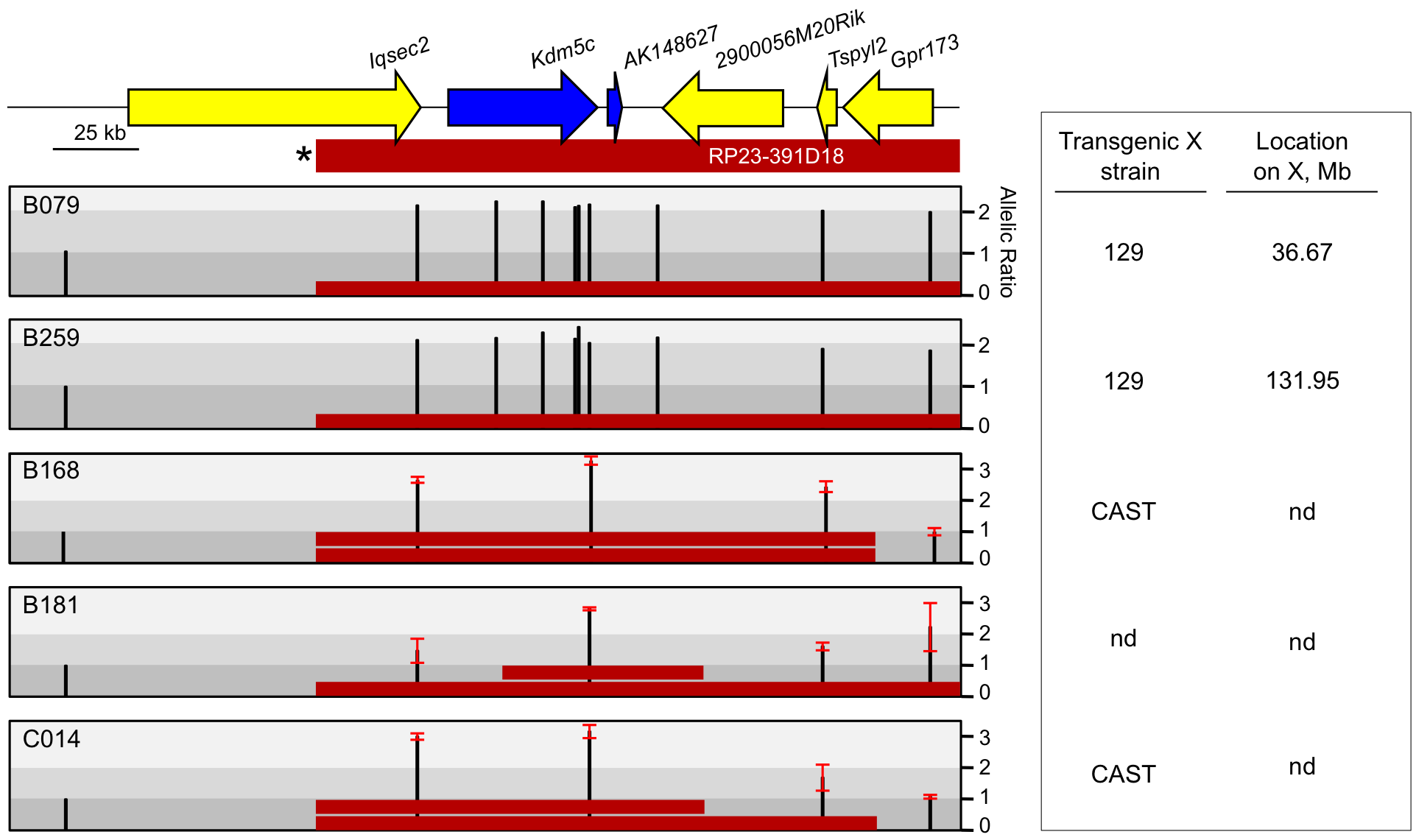

Supplement: Figure S1 — Characterization of additional RP23-391D18-derived transgenes. Transgenes were evaluated and annotated as in Figure 1. Based on SNP ratios, red bar indicates transgene content in each line with the 3′ end drawn at the midpoint of the breakpoint interval. Double bars indicate an increase in transgene copy number. nd: not determined. (TIF) [file pgen.1003952.s001.tif]

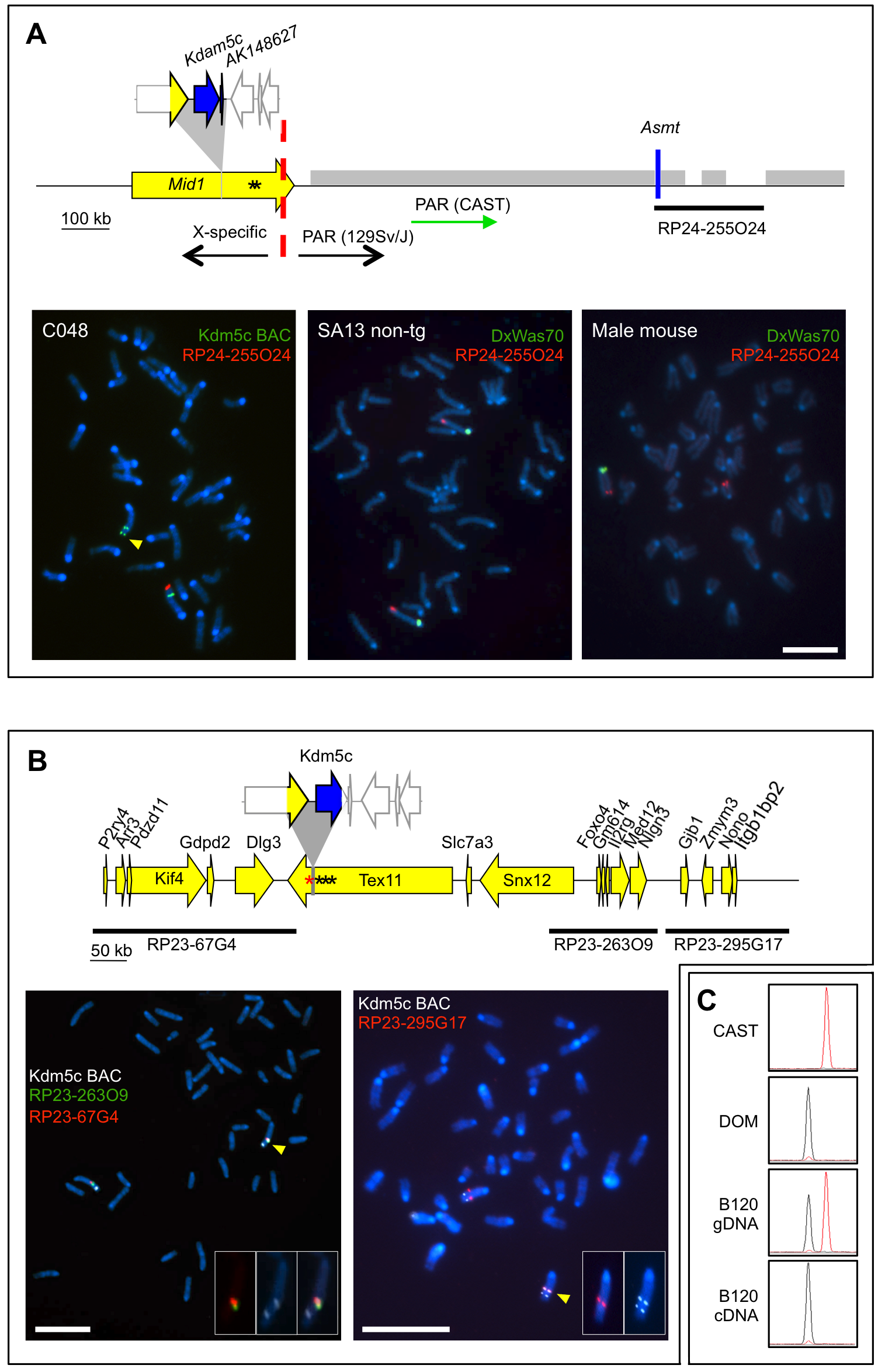

Supplement: Figure S2 — Characterization of transgene integration sites. (A) C048 transgene insertion within the Mid1 gene at Xqter is correctly oriented and to scale, although the transgene inserted on the CAST X chromosome. Available annotated sequence is derived from C57BL/6J (sequence gaps in gray (mm10)). Mid1 spans the pseudoautosomal region (PAR) boundary (dotted line, [55]) in domestic mice, but is X specific in other mouse strains [54]. CAST PAR is distal, but imprecisely defined (green arrow). SNPs indicated by asterisks are deleted on the transgenic CAST X. To identify PAR BACs we screened GenBank high-throughput genomic sequences (HTGS) for clones that partially overlap annotated X and Y sequences. PAR localization of RP24-255O24 was established by FISH with hybridization to both CAST and domestic X chromosomes and to the Y chromosome. Subsequently, RP24-255O24 FISH confirmed PAR deletion on the transgenic C048 X. (B) Gene organization at the C138 integration site drawn to scale. Genomic SNPs (*) as close as 6 kb from the integration site are intact. By FISH, BACs that flank the integration site and the transgene BAC RP23-391D18 are colocalized and rule out large chromosomal alterations. (C) Tex11 is normally X inactivated. Allelic expression of Tex11 SNP rs29083830 (red* in B) was examined in fibroblast line B120 that carries a CAST inactive X. Monoallelic expression from the M.m. domesticus (DOM) active X indicates Tex11 is normally X inactivated. (TIF) [file pgen.1003952.s002.tif]

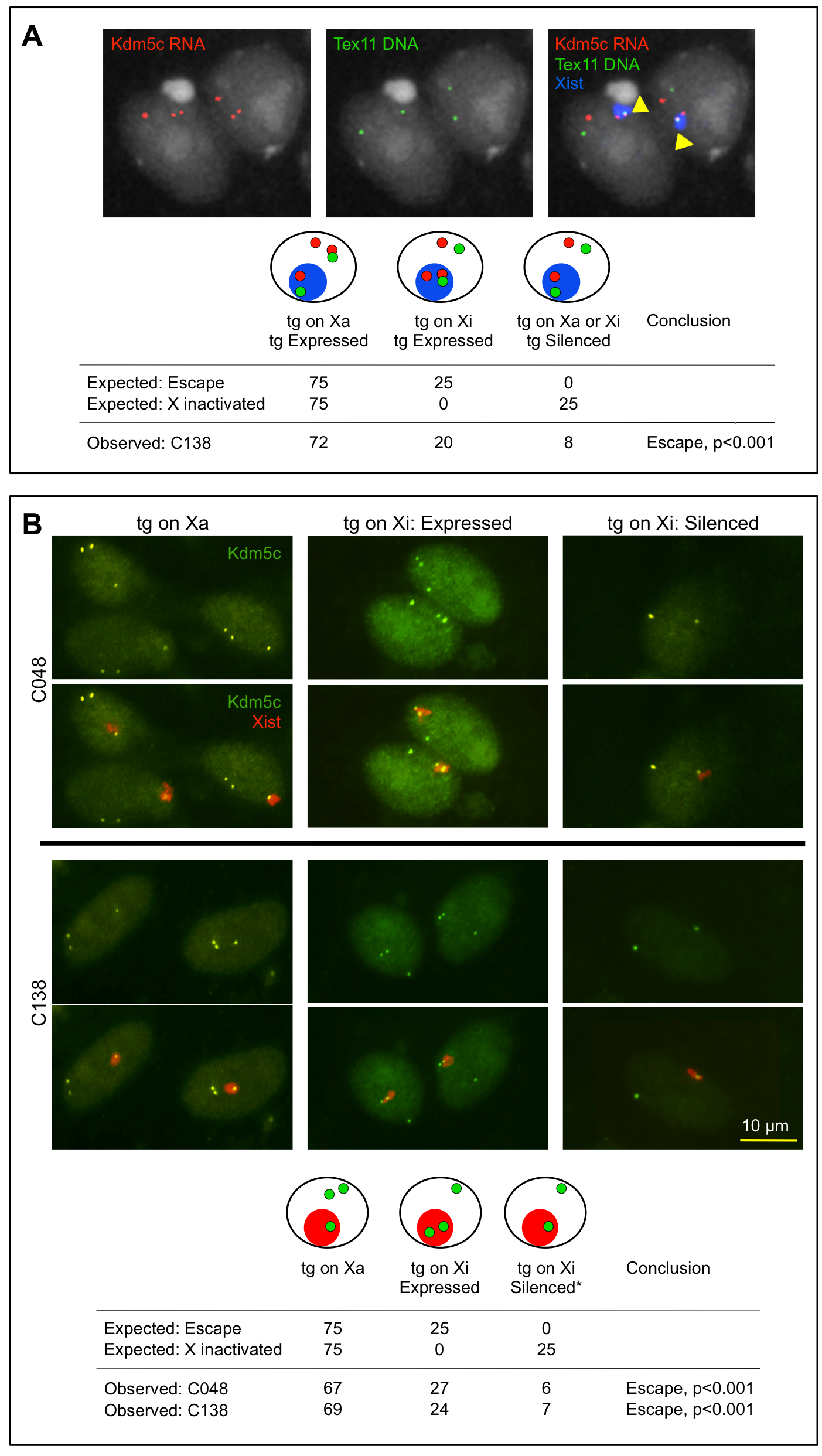

Supplement: Figure S3 — Kdm5c-tg escapes XCI. (A) Sequential RNA and DNA FISH directly demonstrates Kdm5c-tg inactive X expression in C138 by colocalization with an integration site probe. Nuclei were hybridized to detect Xist RNA and Kdm5c RNA (BAC probe) and subsequently denatured and probed for DNA at the integration site (Tex11 BAC). Cartoons represent FISH patterns scored. Results were compared to values expected for Kdm5c-tg to either escape or be X inactivated, as influenced by XCI skewing [19]. n = 50, with cells scored from 37 fields of vision. (B) RNA FISH using a Kdm5c-specific probe establishes Kdm5c-tg escapes XCI. Cells with patterns depicted in cartoons were scored as above. n = 100. Asterisk indicates cells with expression from only a single inactive X allele. We conservatively scored these cells as inactivating Kdm5c-tg, although the inactivated locus could be on either X and derived from either the transgenic or endogenous locus. Both C048 and C138 transgenes escape XCI with results mirroring those in Figure 2. (TIF) [file pgen.1003952.s003.tif]

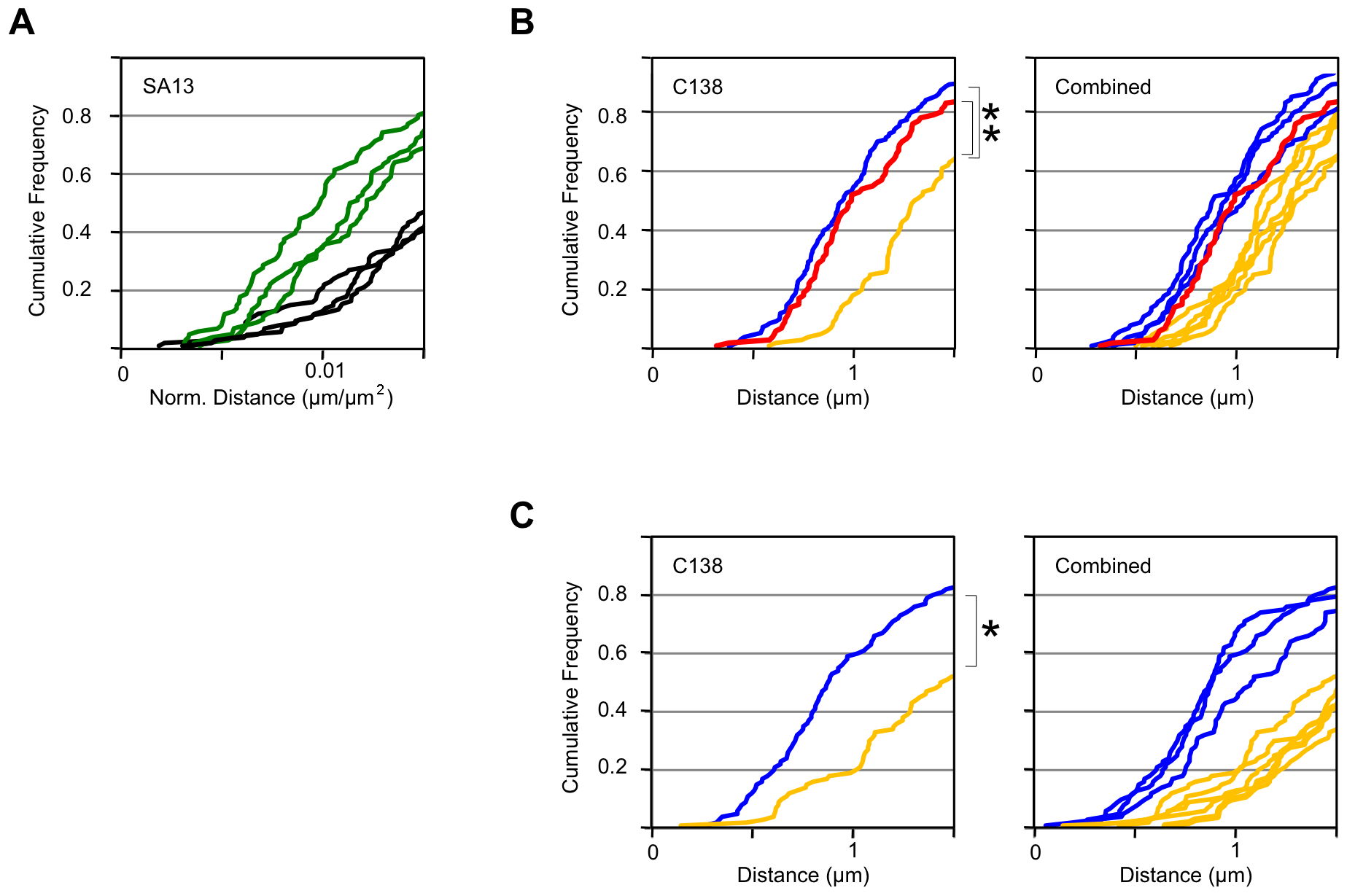

Supplement: Figure S4 — Long-range X-chromosome associations. (A) Ddx3x interactions with distant genes Kdm5c, Tex11, and Mecp2 (as in Figure 3B) differ between active (black) and inactive (green) X chromosomes. Results are shown for the non-transgenic line SA13. (B,C) Interactions with active and inactive loci are apparent regardless of whether distances are normalized to nuclear area. Cumulative frequency plots with non-normalized distances for probe associations with (B) Ddx3x or (C) Kdm5c are shown for C138 or for all comparisons included in the combined plots from Figure 3B and Figure 3C. Colors are as in Figure 3B,C. *p<0.006. (TIF) [file pgen.1003952.s004.tif]

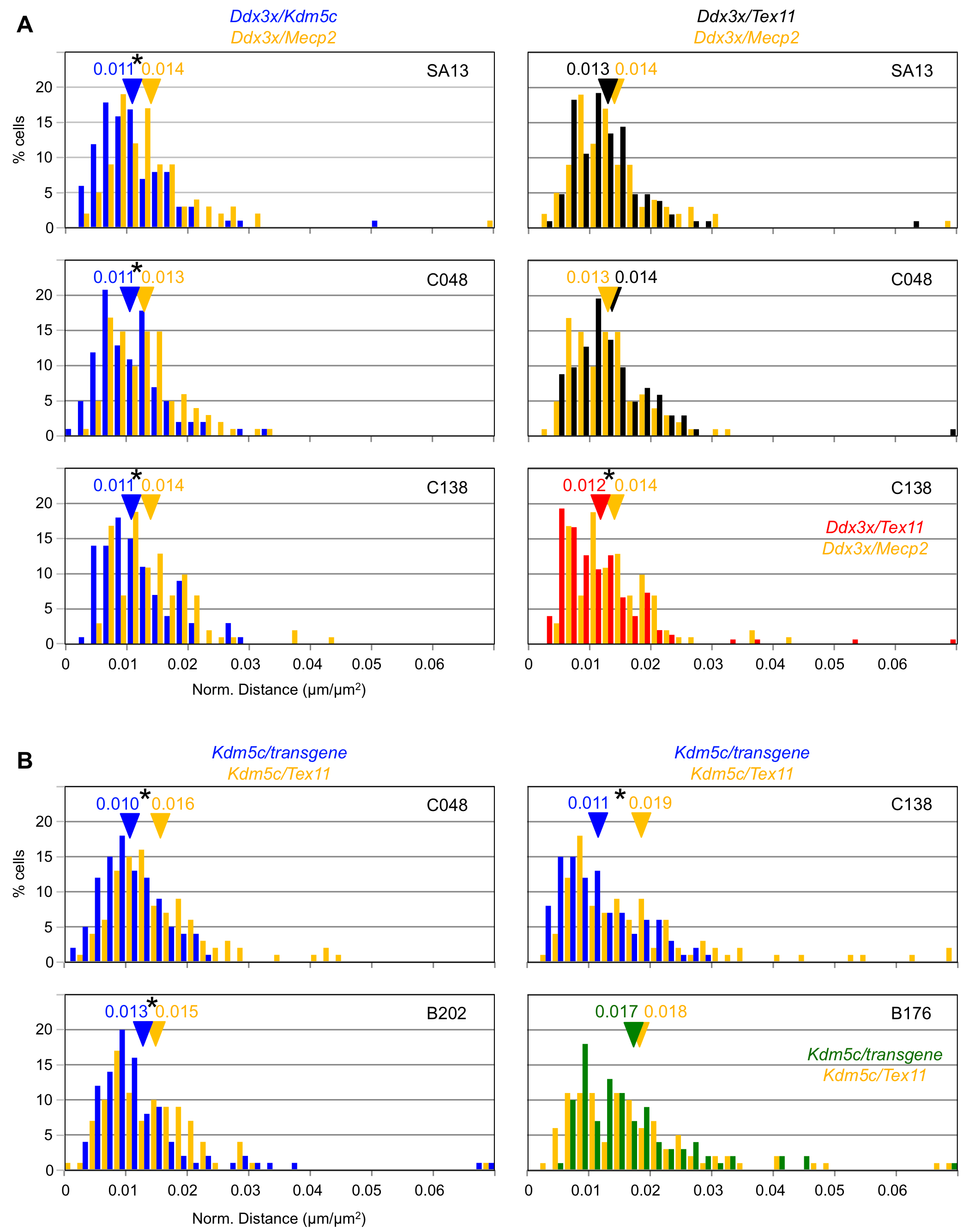

Supplement: Figure S5 — Distribution of complete X-chromosome interaction distances. For all cells scored, normalized distances were binned for probes relative to (A) Ddx3x or (B) Kdm5c. Inverted triangles demarcate average normalized distance. Distributions shifted to the left indicate a larger number of nuclei with probes in close proximity. *p<0.02. (TIF) [file pgen.1003952.s005.tif]

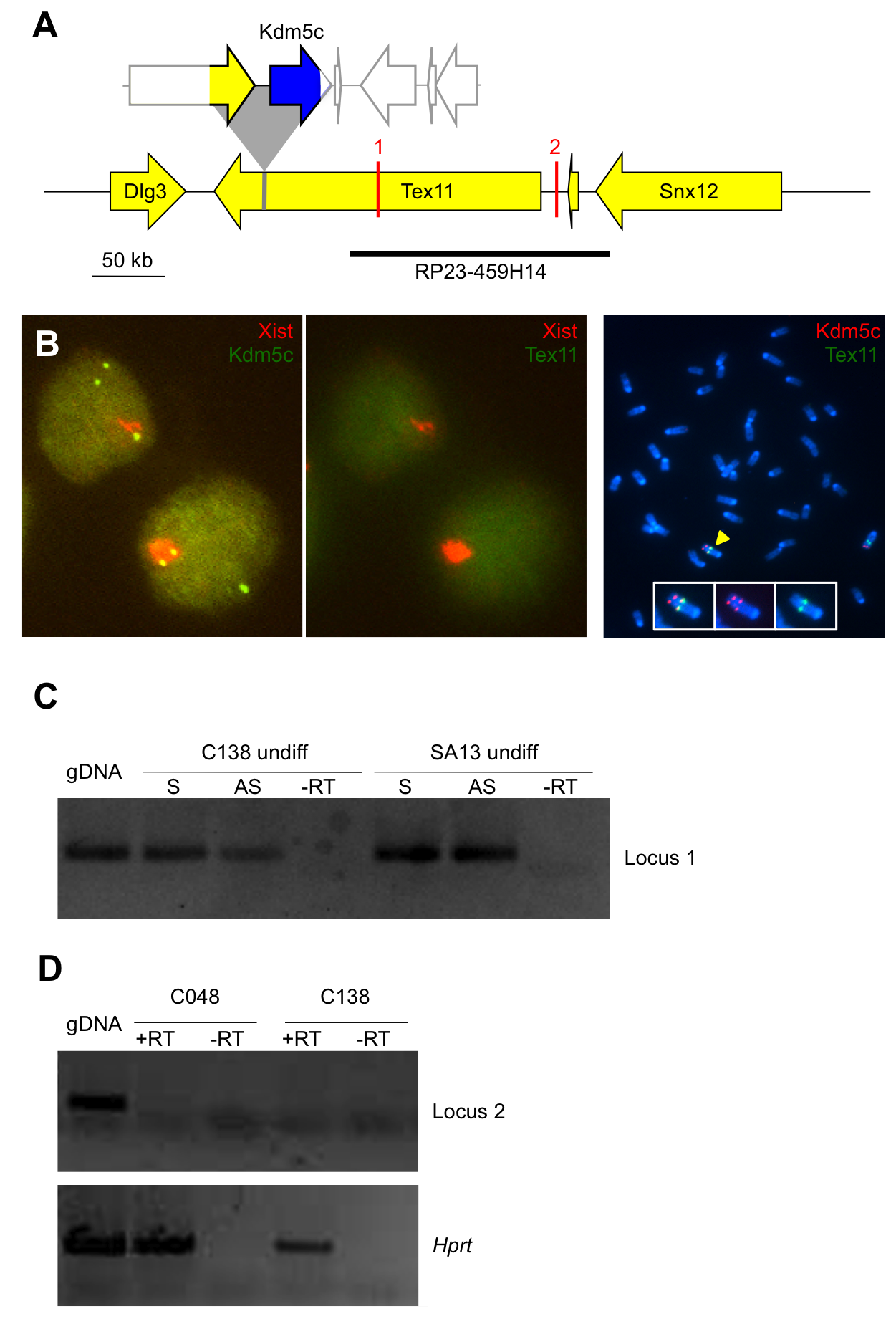

Supplement: Figure S6 — Transcriptional influences at the C138 integration site. (A) Tex11 locus to demarcate FISH probe and RT-PCR amplicons tested. (B) Despite truncation of Kdm5c-tg, read-through transcripts extending across Tex11 are not detected by RNA FISH. Double-stranded BAC probes used for FISH will detect both sense and antisense transcripts. Although robust Kdm5c-tg expression is apparent (first panel, labeled with Alexa594 and pseudocolored green), RNA FISH fails to detect sense (Tex11) or antisense expression (read-through from Kdm5c-tg) using BAC RP23-459H14 (middle panel). DNA FISH is included to demonstrate that the BACs are appropriately labeled. (C) Transcription downstream of Kdm5c-tg appears similar to non-transgenic loci. Strand-specific RT-PCR was performed using cDNA synthesized by reverse transcription with a specific primer. cDNAs were amplified for 35 cycles and low levels of Tex11 (sense (s)) and antisense (as) transcripts were detected (at locus 1 in A) in undifferentiated C138 and non-transgenic (SA13) ES lines. (D) RT-PCR establishes that sequences downstream of Tex11 are not expressed from transgenic or non-transgenic loci (locus 2 in A). (TIF) [file pgen.1003952.s006.tif]

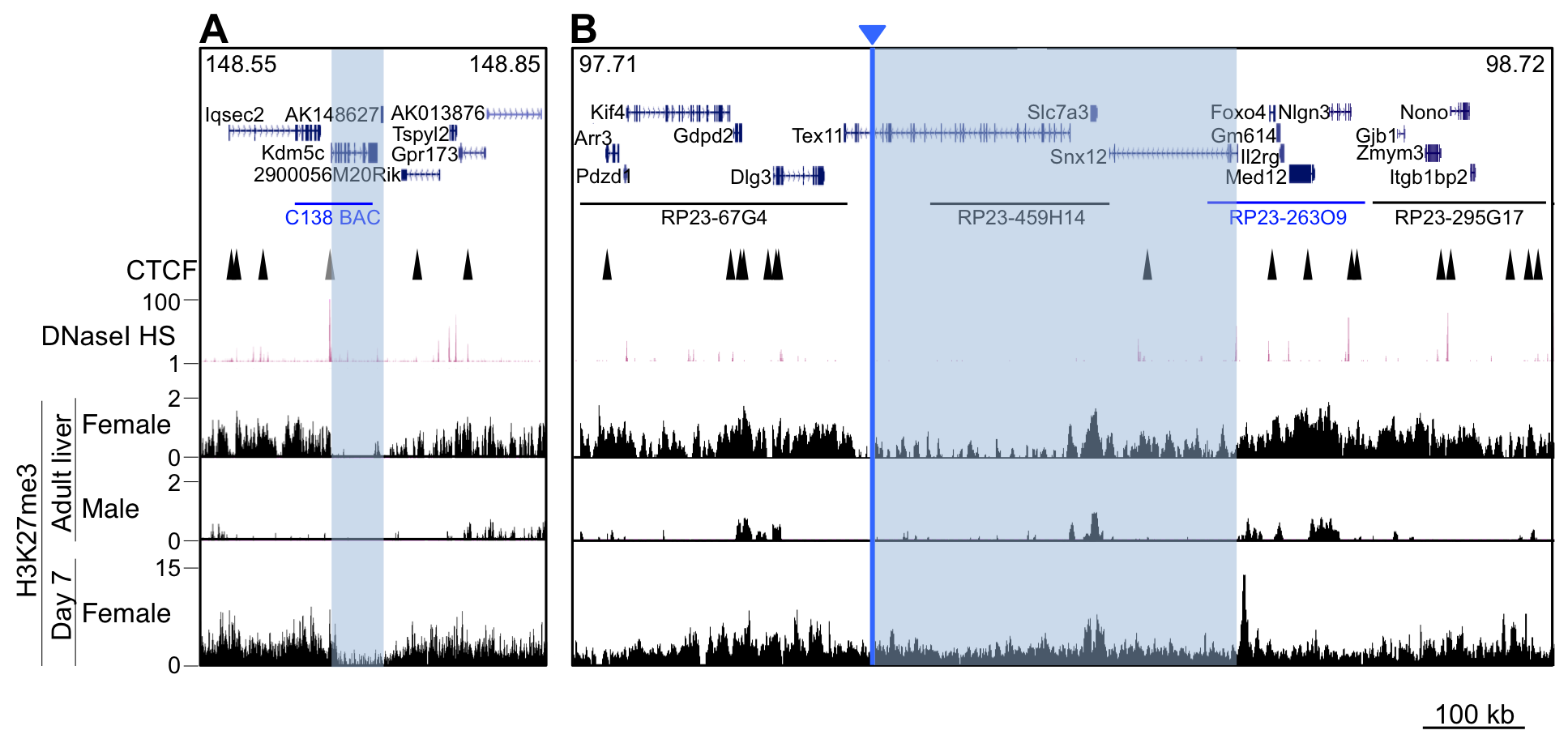

Supplement: Figure S7 — Genomic influences on escape-gene expression. (A) Kdm5c locus with C138 BAC indicated and escapee region shaded. (B) Tex11 locus denoting the C138 integration site (inverted triangle) and aberrant-escape domain (shaded). Genomic coordinates at top are in Mb (mm9). Triangles mark CTCF sites that are conserved in the majority of available data sets (genome.ucsc.edu) (black) or as reported (gray) [24]. DNaseI hypersensitivity is shown for adult female GSM1014171. H3K27me3 occupancy tracks from GSM517917, GSM517918, and GSM905446 are displayed as described in the corresponding references [10], [60]. (TIF) [file pgen.1003952.s007.tif]
