# Supplementary material for: Deletion of an X-Inactivation Boundary Disrupts Adjacent Gene Silencing
Source: PLoS Genet. 2013 Nov 21;9(11):e1003952. doi: 10.1371/journal.pgen.1003952 (PMC3836711; doi:10.1371/journal.pgen.1003952)
Supplement: Table S1 — Oligonucleotide primers. (*) Primer abuts SNP for qSNaPshot. Additional abbreviations: 1Primer amplifies transgenic X, 2Primer amplifies non-transgenic X, 3transgene-specific inverse PCR primer. (PDF) [file pgen.1003952.s008.pdf]

SNP	Location (mm9)	Primer sequence	Experiment
rs29296320	chrX:148,560,583	CACTTGTTGGTCATGCTGCT	Tg breakpoint
		TGCGTAGGATCTCTGCGTTA	
		TCTTGTGGCTATTAAAACAATCTCC*	
rs29296195	chrX:148,619,655	AAATGGAATGGAGTGGGAGTT	
		GCTTTGGTTGTCCTACATACTCA	
		CTACAACCATGTCTTCATAC*	
rs29293592	chrX:148,635,302	TCTGATCTAGGCCCCATGTC	
		CTACTGAGATGCCCCCTGAA	
		TCAGACCCCCTATCTTTACA*	
rs33850225	chrX:148,646,183	AGACAGGAGGGAGGCAGTCT	
		TCGTTTTAGCGATGGGAAAC	
		TGTCTTTTTGCTCAAATAAA*	
rs33850012	chrX:148,658,694	CAGCACTGTGGTCTGAGGAA	
		TCCAGGGACAAGAAAGAGGA	
		GGAAGTGGTAAGAGCCTCTCAG*	
rs8239048	chrX:148,667,548	TGGGCGCGTACTAGTTTAGG	
		CATTGGCGGAAGAACAAAAT	
		GGTAAAAGGATCCGGC*	
rs33850005	chrX:148,661,497	GGACTTCAAAGGCAACCTGA	
		GGCCCACTGTTCATACATCC	
		GACTTGGACAACACCAGCCC*	
Sang20-148662924	chrX:148,662,924	CCAAAATGCCCCTGAAGTTA	
		CCACCACTAACACCGTACAGC	
		TTCTCGTTCCAGAGGACCCA*	
rs8251135	chrX:148,665,594	GCTTGGAAAACACTGAGCAA	
		TGCCTTTCTTTAAGAGCCAAGT	
		TCTGGCTCTGTCACTTTCTA*	
rs8239048	chrX:148,667,548	TGGGCGCGTACTAGTTTAGG	
		CATTGGCGGAAGAACAAAAT	
		GGTAAAAGGATCCGGC*	
rs33848606	chrX:148,680,917	TTCCCGAGGAGATGAAGATG	
		GCAGAACTTGAGGTCCCAAA	
		CTGCCTCCTTTGCCTGAAAT*	
rs33848323	chrX:148,687,198	AGTTTGCAGAGCAGGCCTTA	
		GAGCCTCTTGAAGGCTGAGA	
		CGTACAGGGACTCTTAATAT*	
rs33848201	chrX:148,693,835	GAGACCTGACGCCCTCTTTT	
		GCCAATTTCTCCAAGTTGCT	
		GTTTGTTTCATTTTGATTACCTGATA*	
rs29296613	chrX:148,702,360	GGGGTTCTCTCTCCAACTCC	
		CATGTGTGCCAAAACCACTC	
		GACACAAGATTGAAGTTGAACAGATA*	
rs29296608	chrX:148,703,944	GCTGGGGTGTGTGTGTGTAG	
		AAGATGGTTGCCAGGTTCTG	
		GCTTGTTTCCTGTGCTTCTGA*	
rs29296193	chrX:148,704,803	GAAGGCTGACTGGCTACCTG	
		TCCAAGCAAGGGTAGTGGTC	
		TGGGAAGATGGTCTGCCCT*	
rs29296190	chrX:148,706,085	GCCTCACAGAGAGAGCCATC	
		GGTCACACTGTCCCCATTCT	
		CTGAAGGGTAAGCCAGGGA*	
rs29295457	chrX:148,716,790	GTGACTGGGACCAACAGCT	
		GACAGTGGCAAAACACATGC	
		CTAGCCTATCATTCAGCACTGG*	
rs29295021	chrX:148,725,457	AACTGCTGATCCATCTCTCCA	
		CGGAGGCAGGAGAATCTCTA	
		ACATTTTACTGATGGTATATGTGGATAT*	
rs29294599	chrX:148,729,248	TCAGCCACAAAGAACACAGG	
		TGAATGACTGTGTGACTTCCTTTT	
		CTCAATGTGTCTTTCTACCCAGTAT*	
rs29297026	chrX:148,771,807	TTTGCCCTCCTCTTCAGCTA	
		CCTGACCAAGGCATCACAAT	
		CTCCTCCAAATAAGAACTCTGCA*	
rs29293517	chrX:148,802,031	GGACCTGAATGTGCTCCCTA	
		CACCAAGCAGGTGGGTAGAT	
		TCCAGGGTGAGTGAGCAAA*	

rs29080841	chrX:98,065,382	CACCGAGGTTACTCCGTTCT[1]<br>GAGAAAAGCACAGGGTAGGG[1]<br>TCAGAAGTCTAAGGCCAGCAA[2]<br>CTCTAGGGCCAGAAGCTGGT[2]	Tg X strain, C138
rs31809132	chrX:166,284,547	TCAAAATGGGGCAAGTTCTC[3]<br>AAAGGGGGTGTGTGTGAAAG[3]<br>GACTGCAATACTGGCTGCAA[1,2]<br>GCATTAGGGGAGGAGGAGAA[1,2]<br>GGCTTTTTGGTTACAGGTCAAAG*	Tg X strain, C048
rs29079847	chrX:98,110,584	ATAAATGAATGTACAAATGAAAAGGA<br>CAAGAGCAACTCGGAAACTTTTAG*	Integration site integrity, C138
rs29080319	chrX:98,078,997	CCAACCAGCAAAGTTTTCATT<br>ACTGTGCTTTCTTTGATGGTATC*	
rs29080834	chrX:98,071,608	TGCAAATTTCTGGTATCCAAAG<br>ACAGGTACAATGGGAAATGG*	
rs31818284	chrX:166,365,481	CGCCAACACCATGTCCTC<br>GGTCACACAGTACATATTCACCTTCTC*	Integration site integrity, C048
rs311816672	chrX:166,365,280	CGCCAACACCATGTCCTC*<br>GGTCACACAGTACATATTCACCTTCTC	
rs29083830	chrX:98,146,955	CAAACAAGAAGTCGACTGTCAAGAG<br>TGGAAGCCTTTCCCCACA<br>ATGTTGTGTAACCAATTTAC*	*Tex11* expression
rs31061253	chrX:97,968,357	AGGGAGGATCAGCTCTGGTA*<br>GGCACTCAAAGGAGTGGTCT	*Dlg3* expression
rs29083737	chrX:98,277,423	TTCATCAGCCTGTGTGCTTA*<br>CTCTGCCTGAAGCATTTTCC	*Slc7a3* expression
rs29081924	chrX:98,412,352	CAATCAGGAACTCCAGCTAA*<br>GCAGAACTTGACTGGGGAAG	*Snx12* expression
rs31830290	chrX:98,451,575	TCGCCAGAGAAATACGGACT<br>CAGAAATAGCGCCAAGGGTCT*	*Foxo4* expression
Sang20-98457697	chrX:98,457,697	TCCTAGGCCCACATGATCTC<br>TATTGTGCCCATGCTGAAGA<br>CTTTCTATGTCCTACCTCCC*	*Gm614* expression
Sang20-98463458	chrX:98,463,458	ACTGGACATGAGGACCTTGG<br>CGGAAGCTACGACAAAAGGA<br>CTCCACCCTGCCCTCAGCAG*	*Il2rg* expression
rs29080541	chrX:98,476,172	CTGTGAACTGATCCGACATGA<br>AGGCTTCTGGAAGCTGTGAA*	*Med12* expression
rs29079252	chrX:98,506,295	AAAATGAGGCTAGGCGTGGT<br>CAGATCATGCAGAGACCAGAGA*	*Nlgn3* expression
	Sense *(Tex11)*<br>Anti-sense *to Tex11*<br>*Hprt1*	ATGTTGTGTAACCAATTTAC<br>CAAACAAGAAGTCGACTGTCAAGAG<br>GCCTAAGATGAGCGCAAGTT<br>GTGGGAAAATACAGCCAACACT	Strand specific RT-PCR
	Distal to *Tex11*	ATGTGCCAGTCATGTCCTCA<br>ACATCAGGTGATGCCTCCAT	Read through transcription
